# Sacrificial Biofabrication for Vascularization: Concept, Materials, Technologies, and Applications

**DOI:** 10.1002/adma.202507747

**Published:** 2025-09-09

**Authors:** Jiezhong Shi, Chunyao Wang, Xuan Mei, Ben Zhang, Junxi Wu, Rylie A. Green, Yu Shrike Zhang, Wenmiao Shu

**Affiliations:** ^1^ SINOPEC Key Laboratory of Research and Application of Medical and Hygienic Materials SINOPEC Beijing Research Institute of Chemical Industry Co., Ltd. Beijing 100013 P. R. China; ^2^ Department of Biomedical Engineering University of Strathclyde Glasgow G4 0NW UK; ^3^ Department of Bioengineering Imperial College London London SW7 2BX UK; ^4^ Division of Engineering in Medicine Department of Medicine Brigham and Women's Hospital Harvard Medical School Cambridge MA 02139 USA

**Keywords:** 3D printing, biomedical engineering, hydrogel, sacrificial biofabrication, vasculature

## Abstract

Vasculature plays a crucial role in tissue engineering since it is essential for maintaining tissue viability by efficient nutrient and oxygen exchange as well as waste removal. The creation of biomimetic vascular networks is therefore critical for the development of functional tissue constructs. Sacrificial biofabrication has emerged as an effective method for engineering vascular structures by creating temporary templates that are subsequently removed to form well‐defined vascular channels. In this review, the concept of sacrificial biofabrication is introduced and defined, with a focus on vascularization. Then, a comprehensive overview of the commonly used sacrificial materials based on different types of external stimuli is provided, and the classification and design principles of surrounding materials are briefly introduced. Additionally, various fabrication technologies employed to process sacrificial materials are summarized, and the diverse applications of sacrificial biofabrication in disease models and regenerative medicine are discussed. Finally, the current challenges are highlighted, and the future perspectives for advancing sacrificial biofabrication are explored to address the demand for vasculature manufacturing.

## Graphical abstract

Vasculature is indispensable for tissue viability in regenerative medicine. The sacrificial biofabrication enables precise fabrication of vascular channels by using temporary templates that are subsequently removed. This review defines the concept and delves into sacrificial materials, surrounding materials, fabrication technologies, and biomedical applications, offering insights into current advancements and future prospects in vascular engineering through sacrificial biofabrication.

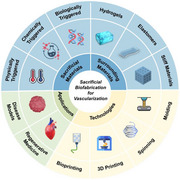

## 1 Introduction

Blood vessels are integral components of the circulatory system, forming an extensive network that facilitates the transport of blood throughout the body.^[^ ^1^ ^]^ Blood vessels are hierarchical networks from the inferior vena cava (15–25 mm) to small capillaries (1–10 µm), which are vital for supplying oxygen and nutrients to cells while removing metabolic wastes, ensuring the functionality of 3D tissues.^[^ ^2^ ^]^ The structure of blood vessels is highly specialized.^[^ ^3^, ^4^ ^]^ Most arteries and veins are composed of three distinct layers: the tunica intima, a smooth endothelial lining that minimizes friction and ensures efficient blood flow; the tunica media, a muscular layer responsible for regulating vascular tone and diameter; and the tunica externa, a connective tissue layer providing mechanical support and elasticity.^[^ ^5^ ^]^ However, in the case of capillaries, they consist only of a single layer of endothelial cells supported by a basement membrane, specifically adapted to facilitate the exchange of substances between the blood and surrounding tissues.^[^ ^4^ ^]^

The construction of blood vessels is crucial in tissue engineering and organ regeneration, especially for engineered thick tissues,^[^ ^6^, ^7^ ^]^ where the absence of internal vascular networks can lead to insufficient oxygen and nutrient delivery,^[^ ^8^ ^]^ resulting in central necrosis over prolonged periods.^[^ ^7^, ^9^ ^]^ A functional vascular system not only sustains the survival and growth of cells but also supports the integration of engineered tissues with host systems upon transplantation.^[^ ^10^ ^]^ Due to the structural complexity, the replication of a perfusable and hierarchical vascular system in vitro still remains a critical challenge.^[^ ^11^ ^]^ One strategy is using decellularised organs as a scaffolding system.^[^ ^12^ ^]^ While these scaffolds retain native vascular structures, their formation requires complex procedures, and the organ shape is uncontrollable. Meanwhile, decellularized organs rely on donor sources, which do not necessarily address the shortage of human organs.^[^ ^13^ ^]^ More importantly, the precise reseeding of the desired cell populations into distinct portions of decellularized organs is also challenging.^[^ ^14^ ^]^ The use of animal‐derived organs further presents significant problems, including the risk of xenobiotic contamination and immunological responses.^[^ ^15^ ^]^ Endothelial pre‐seeding is an effective approach to promote vascularization by seeding endothelial cells onto porous scaffolds before implantation.^[^ ^16^ ^]^ This method allows the endothelial cells to adhere, proliferate, and organize into primitive capillary‐like networks within the scaffolds. These pre‐vascular structures resemble the key features of the native microvasculature and can be further stabilized by co‐cultured stromal cells such as fibroblasts. Upon implantation, host vasculature infiltrates the scaffold and establishes anastomosis with the pre‐formed endothelial networks, leading to the formation of functional and perfusable vessels. This strategy facilitates the rapid vascular integration in vivo, yet the extent of the network maturation and host connection remains difficult to predict and standardize. An alternative strategy for in vitro fabrication of vasculature is based on microfluidic devices.^[^ ^17^ ^]^ utilizing conventional microfabrication technologies^[^ ^18^ ^]^ such as lithography, thin‐film deposition, and etching.^[^ ^19^ ^]^ One approach employs the seeding of endothelial cells within microchannels to generate vasculature,^[^ ^20^ ^]^ but the layer‐by‐layer assembly process is required for the construction of 3D vasculature, which is labor‐intensive and may lead to delamination or structural defects.^[^ ^21^ ^]^ Another approach exploits microfluidic devices^[^ ^22^ ^]^ to mimic the microphysiological environment by perfusing culture medium through two parallel channels. This setup facilitates the assembly of endothelial cells embedded within the hydrogels between the channels into 3D vasculature.^[^ ^23^ ^]^ Nevertheless, the architecture of the generated vasculature cannot be conveniently controlled, and the hydrogels are restricted to naturally derived materials.^[^ ^24^ ^]^

**Figure 1:**
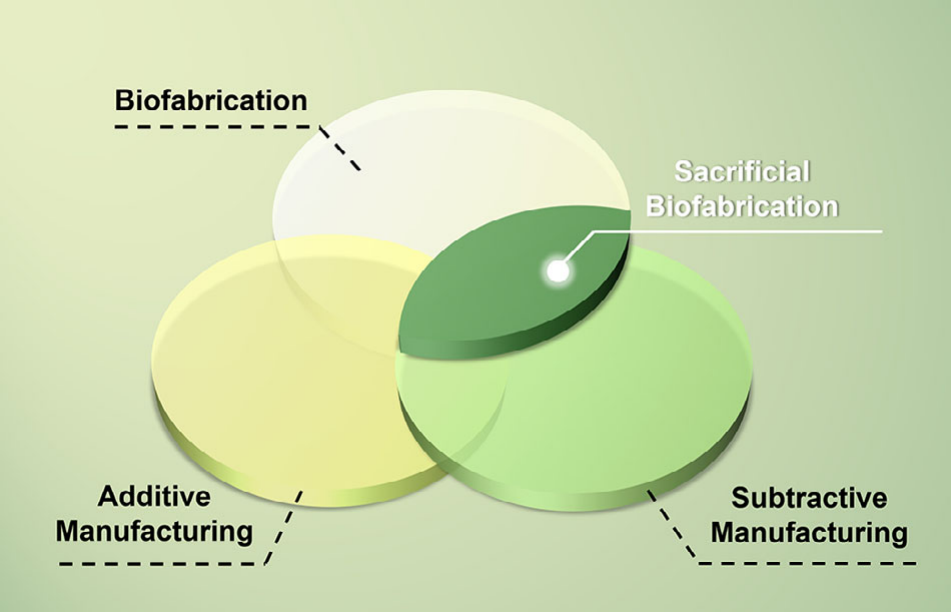
The concept of sacrificial biofabrication in relationship to biofabrication, additive manufacturing, and subtractive manufacturing.

Sacrificial methods are an emerging strategy that has gained significant attention for its ability to overcome many of the abovementioned limitations by introducing temporary, removable templates within tissues to form hollow channels.^[^ ^25^, ^26^ ^]^ While this strategy has been extensively explored for the construction of vasculature in recent years, it has not yet been systematically defined as a unified term. This review introduces the novel term “sacrificial biofabrication” to conceptually organize and unify the existing studies within this growing area of research. Generally, biofabrication denotes the manufacturing process using living cells and biomaterials as fundamental building blocks to develop biologically functional structures.^[^ ^27^, ^28^, ^29^ ^]^ Although biofabrication has emerged as a powerful tool in tissue engineering and regenerative medicine,^[^ ^30^ ^]^ it is often limited by conditions such as insufficient resolution, inadequate porosity, and challenges in constructing intricate channel networks.^[^ ^31^ ^]^ Sacrificial biofabrication integrates subtractive manufacturing and biofabrication—more often supported by additive manufacturing—through the creation and subsequent removal of temporary or sacrificial templates based on selectively pre‐defined patterns (**Figure** **1**), which bridges the gap between structural fidelity and functional complexity. This strategy overcomes the constraints of traditional biofabrication and holds significant potential in engineering vascularized or complex tissues towards applications in both regenerative medicine and in vitro modeling, among many others.

**Figure 2:**
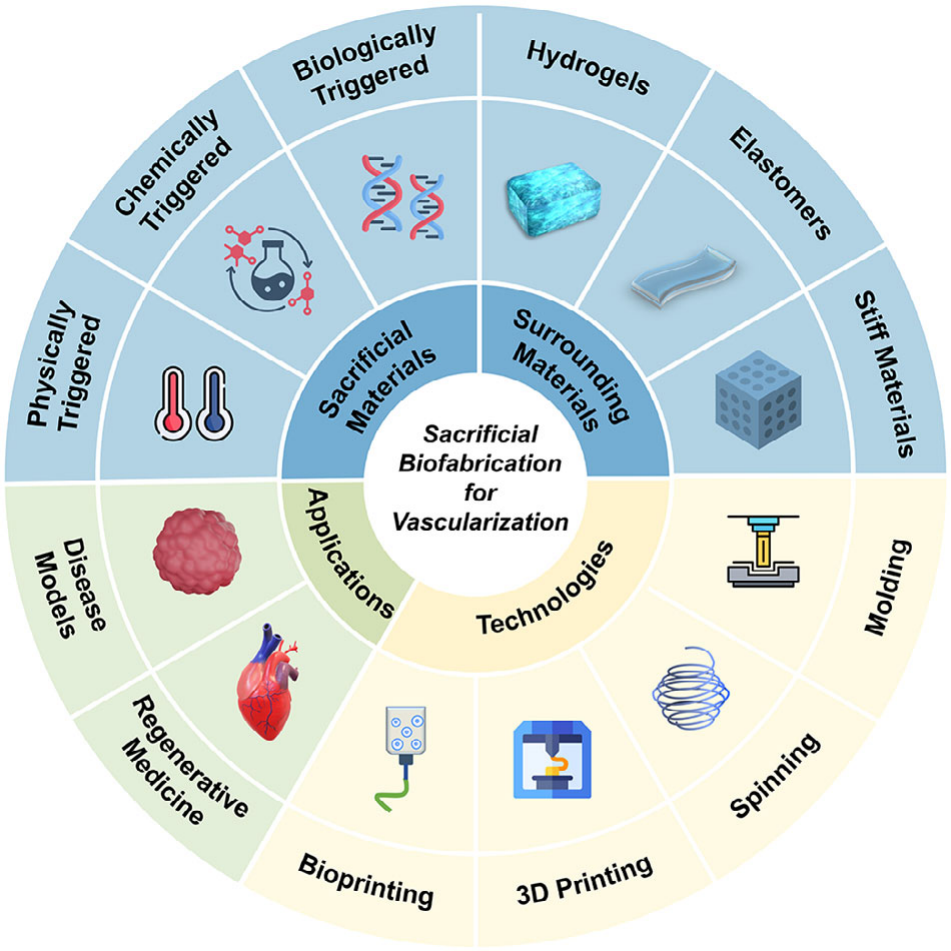
Scheme of materials, technologies, and applications in the sacrificial biofabrication for vascularization.

In this review, the concept of sacrificial biofabrication is first introduced and defined for vascularization. After that, a comprehensive overview of the commonly used sacrificial materials based on physical, chemical, and biological stimuli is provided, and the classification and design principles of surrounding materials are briefly discussed. Then, the main fabrication technologies employed in the sacrificial biofabrication are summarized with a comparison of their pros and cons. The applications of this strategy in disease models and regenerative medicine are also introduced (**Figure** **2**). Finally, current challenges are addressed and insights into the future development of the sacrificial biofabrication are offered.

This review offers a unique perspective that sets it apart from existing reviews.^[^ ^24^, ^25^, ^26^, ^32^, ^33^, ^34^, ^35^, ^36^ ^]^ First, sacrificial biofabrication is a novel term first proposed here, which integrates biofabrication and subtractive manufacturing, frequently with the aid of additive manufacturing, by forming temporary templates and subsequently removing them. Second, most existing reviews mainly introduce the fabrication of vascular‐like channels. In contrast, this review focuses on the preparation of endothelialized vasculature instead of solely on acellular channels since endothelialization is essential for vascular functions. In this review, the diameters of endothelialized vasculature reported in representative studies are uniquely compared, providing valuable insights into the current technological landscape, where key areas for future innovation are also highlighted. Third, in terms of fabrication technologies, this review for the first time extends beyond the conventional focus on 3D printing, offering a holistic overview of all processing techniques employed in sacrificial biofabrication, including molding, spinning, 3D printing, and bioprinting. Notably, a clear distinction between 3D printing and bioprinting in the sacrificial biofabrication is drawn, underscoring the distinct advantages of bioprinting, particularly its ability to circumvent the challenges of uneven cell distribution and vascular occlusion that often arise during post‐printing cell seeding in other technologies. Fourth, unlike other reviews that classify sacrificial materials based on their origins or physical states, this review pioneers a novel categorization according to the different types of external stimuli, dividing them into physically, chemically, and biologically triggered sacrificial materials. This paradigm shift allows to develop a deeper, principle‐driven understanding of sacrificial materials, rather than a purely material‐centric view.

## 2 Concept

**Figure 3:**
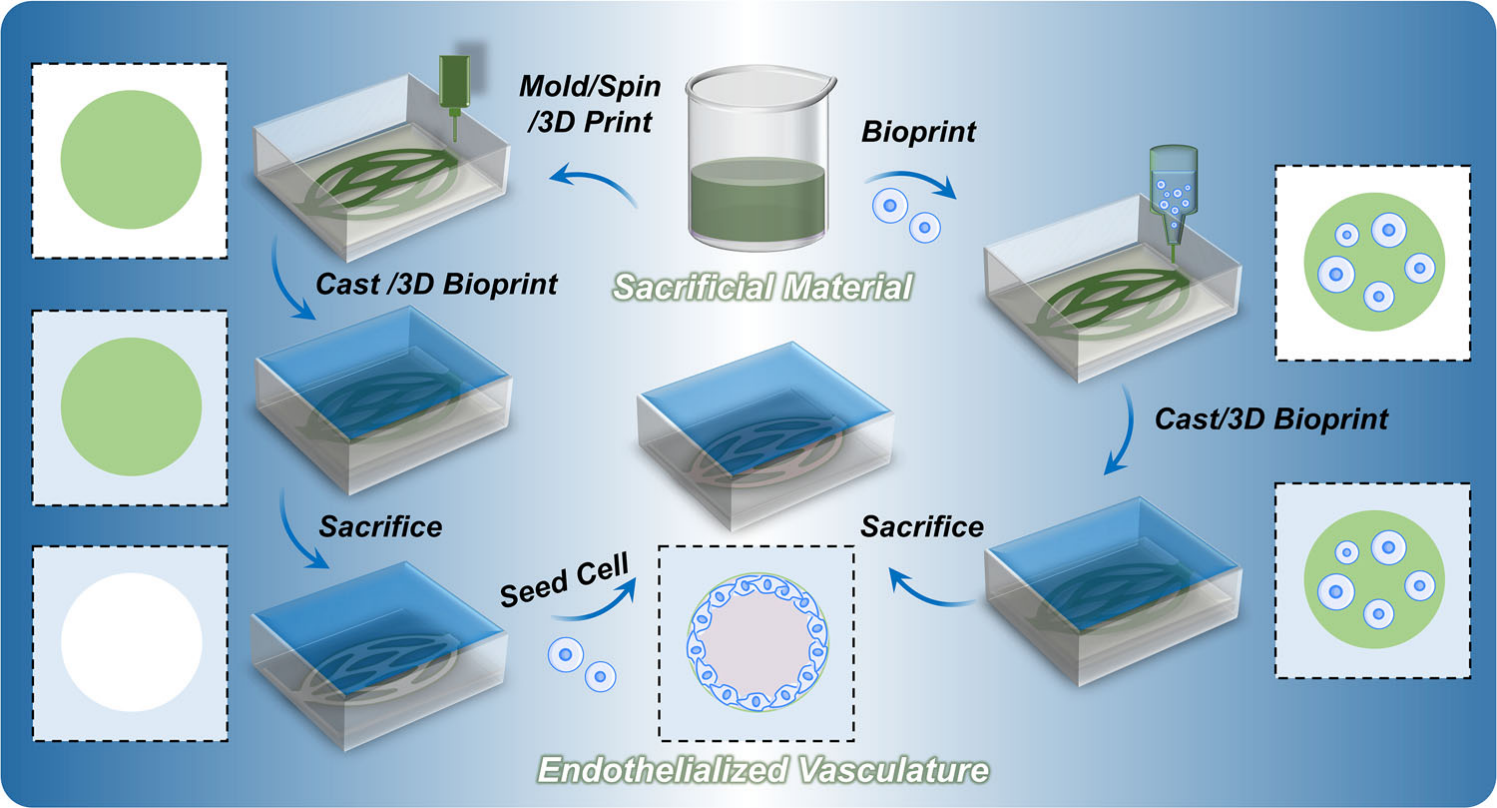
Scheme showing sacrificial biofabrication for vascularization. Endothelialization can be achieved through two methods. Cell seeding in the formed channels (left): endothelial cells are perfused into pre‐formed channels for endothelialization after removal of the sacrificial materials. Cell encapsulation in the sacrificial materials (right): endothelial cells embedded within sacrificial materials spontaneously adhere to the newly formed channel walls upon removal of the sacrificial materials, enabling endothelialization.

Sacrificial biofabrication incorporates biofabrication and subtractive manufacturing, typically with the assistance of additive manufacturing. It can be defined as a biofabrication strategy that creates temporary structures within tissue constructs, which are subsequently removed to form functional spaces such as pores and channels. This strategy was initially demonstrated in 2004 to form cavities in gels,^[^ ^37^ ^]^ and then extended to the fabrication of vascular structures in 2007.^[^ ^38^ ^]^ The typical process of sacrificial biofabrication for vascularization is shown in **Figure** **3**. Sacrificial materials are first processed into specifically shaped filaments, followed by the casting or 3D bioprinting of surrounding materials around them. Upon removal of the sacrificial materials, vascular networks are formed that replicate the geometry of the original filaments.^[^ ^24^, ^32^, ^33^, ^34^, ^35^, ^36^ ^]^ Endothelialization^[^ ^39^ ^]^ can be achieved through two methods. The first involves forming the channels and subsequently seeding them with endothelial cells, which are then perfused to promote endothelialization.^[^ ^40^ ^]^ The second method encapsulates endothelial cells directly within the sacrificial materials.^[^ ^41^ ^]^ As in situ liquification of sacrificial materials occurs under physiological conditions, the embedded cells adhere to the newly formed channel walls, enabling endothelialization. Currently, a series of materials and technologies have been developed in sacrificial biofabrication to prepare vasculature with defined geometries, which show great application potential in biomedical fields.^[^ ^35^ ^]^

## 3 Sacrificial Materials

In the field of tissue engineering and regenerative medicine, sacrificial materials serve as a key factor in the sacrificial biofabrication for the creation of complex vascular structures or channels. Sacrificial materials need to possess sufficient strength to maintain their shapes during the fabrication process, while also being easily removed subsequently without damaging the surrounding scaffolds or tissues. To this end, the success of sacrificial biofabrication largely depends on the selection of the sacrificial materials.

**Figure 4:**
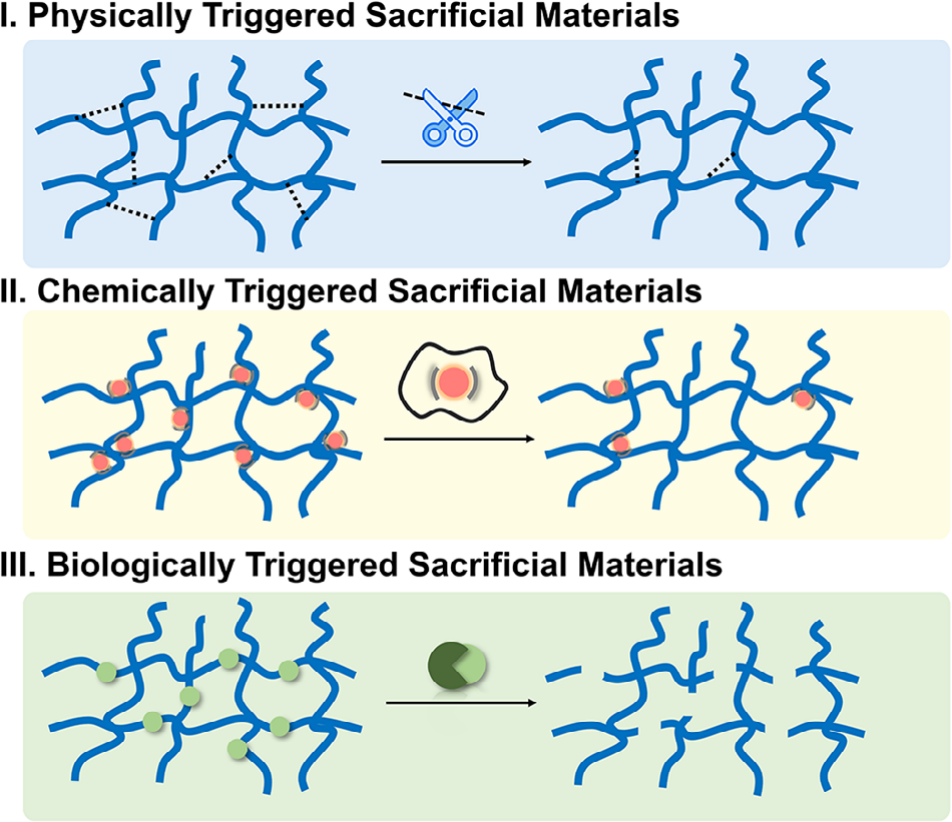
Commonly used sacrificial materials are based on different types of external stimuli. Physical stimuli (temperature, solvents, light, electricity, and capillary forces) and chemical stimuli (pH, chemical reagents) disrupt the intermolecular interactions, leading to material dissolution. Biological stimuli (enzymes) cleave the molecular chains, resulting in material degradation.

There are several existing reviews that have already introduced diverse sacrificial materials,^[^ ^34^ ^]^ which were classified either into natural and synthetic materials by the source of materials, or into hydrogels and polymers by the form of materials.^[^ ^32^ ^]^ In contrast, the sacrificial materials based on different types of external stimuli are primarily categorized into physically triggered sacrificial materials, chemically triggered sacrificial materials, and biologically triggered sacrificial materials (**Figure** **4**). Manual extraction indicates pulling the materials from the surrounding structures by hand or other mechanical means, which is the most traditional removal method. Materials such as stainless steel needles, polyamide fish line, platinum wire,^[^ ^42^ ^]^ agarose,^[^ ^43^, ^44^ ^]^ and polydimethylsiloxane (PDMS)^[^ ^45^, ^46^ ^]^ have been used to create channels through manual extraction. In this review, sacrificial materials refer to those that can be dissolved or degraded under external stimuli rather than physically removed. Therefore, materials based on the physical extraction are not in the scope of this review. Biologically triggered sacrificial materials refer to the materials that can be removed in response to biological stimuli, e.g., enzymes. While biological stimuli can be broadly considered as a subset of chemical stimuli, their high efficiency and specificity warrant separate classification. In the case of materials that can be removed by multiple methods, they are classified under the most widely applied method. This review mainly focuses on the commonly adopted and commercially available materials, whereas customized materials would only be introduced in brief (**Tables** **1** and **2**). Table 1 shows the commonly used sacrificial materials with their fabrication technologies and applications based on different external stimuli. This section primarily focuses on the introduction of the materials, while the technologies and applications will be discussed in detail in the subsequent sections.

**Table 1:** Commonly used sacrificial materials with their fabrication technologies, applications, cyto/biocompatibility, and removal times based on different external stimuli.

External stimuli			Materials	Technologies	Exemplary applications	Cyto/biocompatibility	Reported removal times	Advantages	Disadvantages	Refs.
Physical stimuli	Temperature		Gelatin	Molding, DIW, Embedded printing, Inkjet printing, Bioprinting	Vascular disorder model, Liver tissue model	Excellent	15 min to 2 h	Controllable	Can affect cells	[38, 47, 48, 49, 50, 51, 52]
			Pluronic F‐127	DIW, Co‐axial printing, Embedded printing, Inkjet printing	Glioblastoma model, Neuroblastoma model, CTC behavior, Cancer extravasation,	Good				[53, 54, 55, 56, 57, 58, 59, 60]
			PNIPAM	Solution spinning	Liver tissue model, Skin wound healing	Acceptable				[61, 62, 63]
			PcycloPrOx	MEW	Endothelialized vasculature	Good				[64]
			PIC	DIW	Microchannel	Good				[65]
			HDPC	Solution spinning	Nanofluidic system	Good				[66]
			Petroleum jelly‐liquid paraffin	DIW	Breast tumor model	Good				[67]
			Undecanol‐ethyl lactate	Inkjet printing	Microchannel	Good				[68]
	Solvents	Aqueous media	PVA	Molding, Solution spinning, FDM, Co‐axial printing	Endothelialized vasculature, Tissue construct	Good	10 min to several days	Biocompatible	Slow dissolution	[69, 70, 71, 72]
			Sugar	Melt spinning, FDM	Cardiac patch, Bone regeneration	Good				[73, 74, 75]
				FDM	Angiogenesis, Cardiac tissue model	Good				[76, 77, 78]
			Carbomer	Embedded printing, Co‐axial printing	Vascular tube	Acceptable				[79]
			Carbopol	DIW	Microfluidic device	Acceptable				[80]
			PGF	Melt spinning	Microchannel	Good				[81]
			BVOH	FDM	Tissue flap	Good				[82]
		Organic solvent	PLA	FDM	Macrochannel	Good	5 min to 12 h	Rapid and effective	Toxic to cells	[83]
			PCL	Molding, Melt spinning, Solution spinning, MEW	Sweat gland regeneration	Good				[84, 85, 86, 87]
			PVP/PMMA	Solution spinning	Microchannel	Good				[88]
	Light		Photodegradable hydrogel	Photoablation	Endothelialized vasculature	Good	Minutes to hours	High resolution	Specialized materials	[89]
	Electricity		Cu	/	Microchannel	Poor	2 h	Non‐contact, selective	Conductive materials	[90]
	Capillary forces		Ga	Molding	Multiscalar vasculature	Good	2–5 min	Hierarchical structures	Need precise control	[91]
Chemical stimuli	pH		Shellac	Melt spinning	Microchannel	Good	6–45 h	Controllable	Potential cytotoxicity	[92, 93]
			Alkali‐soluble photopolymer	SLA	Microchannel	Good				[94, 95]
	Chemical reagents		Alginate	Molding, DIW, Embedded printing,	Adipose tissue model	Excellent	0.5–4 h	Rapid and effective	Potential cytotoxicity	[96, 97, 98]
			PEC	Embedded printing	Subcutaneous connective tissue regeneration	Good				[99]
			Dual‐component hydrogel	Embedded printing	Cardiac tissue model	Good				[100, 101]
			Synthetic self‐healing hydrogel	DIW	Endothelialized vasculature	Good				[102]
			Thioester‐based elastomer	DLP	Patterned void space	Acceptable				[103]
Biological stimuli	Enzymes		HA	Bioprinting	Endothelialized vasculature	Excellent	0.5–20 h	Highly specific	Expensive and slow	[104]
			DNA	Bioprinting	Liver tissue model	Excellent				[41]

Abbreviations: BVOH: butanediol vinyl alcohol copolymer; CTC: circulating tumor cell; Cu: copper; DIW: direct ink writing; DLP: digital light processing; DNA: deoxyribonucleic acid; FDM: fused deposition modeling; Ga: gallium; HA: hyaluronic acid; HDPC: heat depolymerizable polycarbonate; MEW: melt electrospinning writing; PCL: polycaprolactone; PcycloPrOx: poly(2‐cyclopropyl‐2‐oxazoline); PDMS: polydimethylsiloxane; PEC: polyelectrolyte complexes; PGF: phosphate‐based glass fibers; PIC: polyIsoCyanide; PLA: polylactic acid; PMMA: poly (methyl methacrylate); PNIPAM: poly(*N*‐isopropylacrylamide); PVA: polyvinyl alcohol; PVP: poly (vinyl pyrrolidone); SLA: stereolithography

**Table 2:** Representative examples of materials and technologies used in the sacrificial biofabrication for vascularization.

Technologies			Sacrificial materials	Surrounding materials	Removal stimuli	D_v_ [µm]	D_e_ [µm]	General advantages	General disadvantages	Refs.
Molding			Gelatin	Collagen/ Fibrinogen/ Matrigel	Temperature	6	50	High resolution	Time‐consuming, 2D	[38]
			Alginate	Gelatin/ Collagen/ Agarose	EDTA	20	200			[96]
			PVA	HEMA/ GelMA/ Agarose	Aqueous media	100	300			[69]
			PCL	Collagen	Acetone	50	50			[84]
			Gelatin	Alginate	Temperature	1000	1000			[171]
			Ga	Agarose/ Collagen/ Fibrin	Capillary forces	30	39			[91]
Spinning	Melt spinning		Sugar	PDMS	Aqueous media and ethanol	1	/	High resolution	Uncontrollable shape, high temperatures	[73]
			Sugar	PDMS	Aqueous media and ethanol	10	/			[172]
			Shellac	Gelatin	pH	10	/			[92]
			Shellac	Collagen	Ethanol and PBS	10	10			[93]
	Solution spinning		PNIPAM	Gelatin	Temperature	3	/	High resolution	Uncontrollable shape, solvents toxicity	[61]
			PVA	PGS	Aqueous media	150	/			[70]
			PCL	Gelatin	Dichloromethane	2	/			[85]
3D printing	Extrusion‐based printing	FDM	Carbohydrate glass	Agarose/ Alginate/ PEG/ Fibrin/ Matrigel	Aqueous media	200	200	Low cost, versatile	High temperature, Require filament‐based materials	[76]
			PVA	Gelatin	Aqueous media	1300	1300			[71]
		DIW	Pluronic F‐127	GelMA	Temperature	100	200	Simple, versatile	Limited complexity	[53]
			Pluronic F‐127	Gelatin	Temperature	100	200			[173]
		Co‐axial printing	Pluronic F‐127	VdECM and alginate	Temperature	500	500	Multi‐layers	Complex setup, layer adhesion	[54]
			PVA	GelMA	Aqueous media	251	610			[72]
			Pluronic F‐127	Collagen and alginate	Temperature	1300	1300			[174]
		Embedded printing	Pluronic F‐127	Diacrylate‐functionalized Pluronic F‐127	Temperature	18	/	Suspended and complex structures	Rely on support bath	[55]
			Dual‐component hydrogel	AdNor‐HA	β‐cyclodextrin	500	500			[100]
			Alginate	Agarose/ Gelatin/ GelMA	Sodium citrate	200	1000			[97]
			Carbomer	Bioelastomer	PBS	300	500			[79]
			Gelatin	OBB matrices	Temperature	400	500			[47]
			Gelatin	Collagen	Temperature	125	500			[175]
	Light‐based printing	SLA	Alkali‐soluble photopolymer	PLGA/ PCL/ PLA/ Chitosan/ Alginate/ Bone cement	pH	50	/	High resolution, smooth	Slow, post‐processing	[94]
			Alkali‐soluble photopolymer	PEGDA	pH	80	/			[95]
		DLP	Thioester‐based elastomer	Matrigel/ PEG	2‐Mercaptoethanol	22	/	Fast, smooth	Limited resolution, expensive	[103]
	Inkjet printing		Gelatin	Fibrin and collagen	Temperature	1000	1000	High resolution	Limited material options	[48]
			Pluronic F‐127	GelMA	Temperature	30	30			[56]
	SLS		Carbohydrate	PDMS/ PCL/ PEGDA/ Agarose/ Silk fibroin/ Fibrin	Aqueous media	300	300	Suspended and seamless branched structures	Low resolution	[176]
	MEW		PCL	HAMA	Acetone and dichloromethane	10	300	High resolution, controlled fiber alignment	High temperature, high Voltage	[86]
			PcycloPrOx	GelMA	Temperature	110	400			[64]
Bioprinting	DIW		Gelatin	Collagen	Temperature	/	500	Live cell printing without perfusion	Limited bioink	[49]
	DIW		Gelatin	GelMA	Temperature	/	450			[177]
	Co‐axial printing		Gelatin	GPT	Temperature	/	485			[178]
	Co‐axial printing		Gelatin	GelMA	Temperature	/	200			[179]
	DIW		DNA	GelMA	Exonuclease	/	70			[41]
	Inkjet printing		HAMA	GelMA	Hyaluronidase	360	360			[104]

Abbreviations: 2D: two‐dimensional; 3D: three‐dimensional; AdNor‐HA: adamantane and norbornene modified hyaluronic acid; D_e_: the smallest diameter of endothelialized vasculature; DIW: direct ink writing; DLP: digital light processing; DNA: deoxyribonucleic acid; D_v_: the smallest diameter of acellular vascular structure; EDTA: ethylene diamine tetraacetic acid; FDM: fused deposition modeling; GelMA: gelatin methacrylate; GPT: gelatin‐PEG‐tyramine; HAMA: hyaluronic acid methacrylate; HEMA: 2‐hydroxyethyl methacrylate; MEW: melt electrospinning writing; OBB: organ building block; PBS: phosphate buffered saline; PCL: polycaprolactone; PcycloPrOx: poly(2‐cyclopropyl‐2‐oxazoline); PDMS: polydimethylsiloxane; PEG: polyethylene glycol;PEGDA: polyethylene glycol‐diacrylate; PLA: polylactic acid; PLGA: poly(lactic‐co‐glycolic) acid; PNIPAM: poly(N‐isopropylacrylamide); PGS: poly(glycerol sebacate); PVA: polyvinyl alcohol; SLA: stereolithography; SLS: selective laser sintering; TPP: two‐photon polymerization; VdECM: vascular‐tissue‐derived decellularized extracellular matrix.

### 3.1 Physically Triggered Sacrificial Materials

Physically triggered sacrificial materials refer to the materials that can be removed by physical stimuli, such as temperature, solvents, light, electricity, and capillary forces. These stimuli are usually mild and biocompatible, avoiding the potential toxicity of chemical substances.

#### 3.1.1 Temperature

Temperature‐based removal leverages the thermo‐responsive behavior of certain materials, prompting their transition into a liquid state when exposed to specific temperatures. This method is highly controllable, allowing for precise manipulation of material states by simply adjusting the temperature. This precision is particularly advantageous for creating fine, uniform channels in complex structures. Nonetheless, the primary disadvantage arises from the potential negative impact on biological systems. Rapid or significant temperature changes can damage cells or sensitive biological components, which limits the applications of this method in systems where maintaining cell viability is critical. Among the temperature‐responsive materials, a variety of natural and synthetic polymers have been utilized as sacrificial materials, each with distinct gelation and removal temperatures, leading to varied degrees of biocompatibility and controllability.

Gelatin is a natural biopolymer derived from collagen, which has excellent biocompatibility due to the presence of Arg‐Gly‐Asp (RGD) sequence for cell adhesion.^[^ ^105^, ^106^ ^]^ It forms a hydrogel below its gelation temperature (≈30–35 °C) through the reassembly of triple‐helical structures. Upon heating above the gelation temperature, the helices dissociate, resulting in hydrogel dissolution and enabling its removal through thermal treatment.^[^ ^38^ ^]^ It is notable that gelatin can be dissolved under physiological conditions without harming cells, which allows it to encapsulate cells to aid cell seeding even when used as a sacrificial material.

Pluronic F‐127 is an amphiphilic triblock copolymer of poly(ethylene oxide) (PEO) and poly(propylene oxide) (PPO).^[^ ^107^, ^108^ ^]^ Above its critical gelation temperature (10–20 °C), the PPO segments aggregate, leading to micelle formation and the establishment of gel networks stabilized by the hydrophilic PEO chains. When the temperature is reduced, the micelle breaks apart and the hydrogel is liquified, which allows for its removal via cooling (usually at 4 °C).^[^ ^53^ ^]^ While Pluronic F‐127 offers superior printability compared to gelatin, its reliance on low‐temperature removal and associated cytotoxicity pose significant challenges for use in cell‐laden constructs.

Poly(*N*‐isopropylacrylamide) (PNIPAM) is a thermoresponsive polymer with a lower critical solution temperature (LCST) of ≈32 °C.^[^ ^109^ ^]^ Above the LCST, PNIPAM exhibits hydrophobicity and insolubility in aqueous media, maintaining its structural integrity. Conversely, when the temperature drops below its LCST, PNIPAM undergoes a sharp phase transition to a hydrophilic state, resulting in its complete dissolution in aqueous media.^[^ ^110^ ^]^ This reversible thermoresponsive behavior makes PNIPAM an ideal sacrificial material, which can be removed at room temperature.^[^ ^61^ ^]^ Similar to PNIPAM, poly(2‐cyclopropyl‐2‐oxazoline) (PcycloPrOx) is a synthetic polymer with a LCST of 25 °C. Thus, it can retain its shape at temperatures above 25 °C and be removed by dissolving in aqueous media below 25 °C.^[^ ^64^ ^]^ However, both PNIPAM and PcycloPrOx lack inherent bioactivity, which may complicate their use in cell‐laden systems. PolyIsoCyanide (PIC) hydrogel transitions from a soluble state at low temperatures (≤18 °C) to a stable gel at physiological temperature (37 °C). To remove PIC hydrogel, the structure is washed with cold phosphate‐buffered saline (PBS) at 4 °C, causing the hydrogel to revert to a liquid state and diffuse out.^[^ ^65^ ^]^

In addition, heat‐depolymerizable polycarbonate (HDPC) is synthesized through the reaction of cyclohexene oxide and carbon dioxide with a zinc‐based catalyst, which can be depolymerized and removed above 250 °C.^[^ ^66^ ^]^ The mixture of petroleum jelly and liquid paraffin is hydrophobic and temperature‐responsive, which can be used as sacrificial ink and removed by liquifying it at 70 °C.^[^ ^67^ ^]^ Undecanol and ethyl lactate at the mass ratio of 3:1 can be used as a liquid sacrificial template and removed by evaporation at 90 °C.^[^ ^68^ ^]^ These materials require significantly elevated temperatures for removal, which imposes substantial constraints on their use in biologically relevant or temperature‐sensitive applications.

#### 3.1.2 Solvents

Commonly used solvents typically include aqueous media and organic solvents. Aqueous medium‐based removal utilizes the solubility of sacrificial materials in aqueous media, making it a biocompatible option by avoiding the use of harmful chemicals. This method minimizes the risk to cells and tissues, which is highly suitable for applications in tissue engineering. However, its major disadvantage is the slow dissolution rate of materials in aqueous media, a factor that can greatly extend the overall process time. For larger or denser materials, multiple washes may be required to ensure complete removal, adding further delays to the fabrication process.

Polyvinyl alcohol (PVA) is a synthetic, water‐soluble polymer widely used in biomedical fields owing to its mechanical stability and biocompatibility.^[^ ^111^ ^]^ PVA can be processed into filaments at temperatures between 180–220 °C, where it transits into a thermoplastic state without significant degradation.^[^ ^112^ ^]^ After use, PVA filaments can be dissolved in aqueous media at room temperature or slightly elevated temperatures (40–50 °C), allowing for removal as sacrificial material.^[^ ^69^ ^]^

Sugar, encompassing simple saccharides like glucose, sucrose, and trehalose, serves as a highly versatile and biocompatible material^[^ ^113^ ^]^ known for its ease of processing and water solubility.^[^ ^73^ ^]^ It can be processed when the temperature increases to 100–150 °C, where it transitions into a molten state suitable for extrusion or shaping. Carbohydrate glass, made from sugars or sugar derivatives, is an amorphous material with great biocompatibility and biodegradability.^[^ ^114^ ^]^ It can also be 3D‐printed at temperatures ranging from 100–150 °C.^[^ ^76^ ^]^

Additionally, carbomer is the synthetic polymer of polyacrylic acid with high molecular weight,^[^ ^115^ ^]^ which can be removed by flushing with water or buffers like PBS.^[^ ^79^ ^]^ Carbopol is a registered trademark for specific grades of carbomer.^[^ ^116^ ^]^ It is one of the most widely used commercial brands of carbomers.^[^ ^80^ ^]^ Phosphate‐based glass fibers (PGF), composed mainly of biodegradable phosphate and calcium compounds, can be degraded in aqueous media with adjustable time from minutes to years via chemical modification.^[^ ^81^ ^]^ Butanediol vinyl alcohol copolymer (BVOH) is a water‐soluble material commonly used in 3D printing to create sacrificial templates, which can be removed by dissolution in water.^[^ ^82^ ^]^

On the other hand, organic solvent‐based removal employs solvents to dissolve sacrificial materials that are more hydrophobic. This method is rapid and effective, often allowing for complete removal in a short time. However, organic solvents can introduce toxicity, particularly in biological environments, which makes them less suitable for applications involving living cells. Thus, thorough post‐removal washing is necessary to eliminate any residual solvents.

Polylactic acid (PLA)^[^ ^117^ ^]^ and polycaprolactone (PCL)^[^ ^118^ ^]^ are two biodegradable thermoplastic polymers with significant applications in biomedical, packaging, and engineering fields.^[^ ^119^ ^]^ Both polymers are commonly processed using conventional thermoplastic approaches, including extrusion, injection molding, and 3D printing. PLA requires processing temperatures in the range of 180–220 °C,^[^ ^120^ ^]^ while PCL may be processed at 60–120 °C,^[^ ^121^ ^]^ offering energy efficiency and reduced risks of thermal degradation. Following their applications, PLA and PCL can be removed effectively by dissolution in organic solvents, such as chloroform, dichloromethane, acetone, and toluene.^[^ ^83^, ^84^ ^]^ Poly(vinyl pyrrolidone)/poly(methyl methacrylate) (PVP/PMMA)^[^ ^122^ ^]^ integrates the mechanical support of PMMA and the hydrophilicity of PVP, and it can be dissolved in chloroform or toluene to remove.^[^ ^88^ ^]^

#### 3.1.3 Light

Light‐mediated sacrificial material removal relies on precise photodegradation, where targeted illumination induces bond cleavage, enabling controlled material evacuation​. This approach offers exceptional spatial resolution and enables on‐demand, programmable degradation with cytocompatibility, even in the presence of live cells. However, the limited light penetration constrains its scalability. Moreover, the process requires specialized photosensitive materials, restricting its versatility in certain biomedical applications.

A photodegradable hydrogel is synthesized through strain‐promoted azide‐alkyne cycloaddition between a diazide‐functionalized synthetic peptide and polyethylene glycol (PEG)‐tetrabicyclononyne.^[^ ^89^ ^]^ The peptide crosslinker incorporates ortho‐nitrobenzyl (oNB) moieties, which are photoresponsive to near‐infrared light (740 nm) and undergo multiphoton‐assisted photolysis. Upon light exposure, the oNB groups absorb photons, initiating a photochemical reaction that breaks the crosslinking bonds within the hydrogel network, leading to localized material degradation under precisely defined optical conditions. Following photodegradation, the resulting soluble sacrificial material can be removed from the fabricated channels by diffusion or perfusion with cell culture medium.

#### 3.1.4 Electricity

The removal of sacrificial materials using electricity is based on electrolysis, where an applied electric current selectively dissolves the materials. This process commonly uses metals as the anode in electrolyte solutions. When electricity is applied, metals undergo oxidation, breaking down into ions and dissolving into the solution. This method offers non‐contact processing, which makes it ideal for applications requiring minimal mechanical stress. Additionally, its ability to selectively remove sacrificial materials is highly beneficial in microfabrication and precision engineering. Nonetheless, it is limited to conductive materials and requires careful electrolyte management. Meanwhile, the electrolysis process is slow, and electrical stimulation may cause damage to cells when used in the biomedical field.

Copper (Cu) has been used as a sacrificial material to form channels. In this case, a Cu wire was initially immersed and electrolyzed in an alginate solution, during which Cu^2+^ was released from the wire surface and crosslinked nearby alginate molecules to form a thin alginate hydrogel coating around the Cu wire.^[^ ^90^ ^]^ The gel‐coated wire was subsequently immersed a calcium chloride (CaCl_2_) solution for further electrolysis to remove most of the Cu. After fully dissolving the remaining Cu in a ferric chloride (FeCl_3_) solution, a channel was finally formed within the alginate hydrogel. Nonetheless, the use of copper raises concerns due to the toxicity of residual Cu^2^⁺ ions, which may limit its applicability in biological systems.

#### 3.1.5 Capillary Force

Capillary force enables the removal of sacrificial materials by leveraging surface tension and Laplace pressure differentials to drive liquid evacuation. This method allows for stress‐free extraction, preserving delicate biological structures while achieving multiscalar fabrication, which seamlessly integrates both large‐scale and micro‐scale features, such as hierarchical vascular networks. However, precise control of capillary pressure is essential to ensure complete removal since excessive surface tension gradients can cause fragmentation. Furthermore, solvent composition and gel elasticity have to be optimized to avoid unintended deformation of sacrificial templates.

Gallium (Ga) is a special type of metal with a low melting point of 29.8 °C and unique surface properties that make it an ideal sacrificial material.^[^ ^123^ ^]^ As a sacrificial template, Ga is a resilient solid at room temperature. To remove it, Ga is first heated to 32 °C to melt into a liquid, and then geometric asymmetry is created and native oxide layer is dissolved by sodium hydroxide (NaOH) to generate surface tension gradients and Laplace pressure differentials, which drives the directional flow Ga without damaging the surrounding soft matrix until it is fully evacuated.^[^ ^91^ ^]^

### 3.2 Chemically Triggered Sacrificial Materials

Chemically triggered sacrificial materials indicate the materials that can be removed through chemical stimuli, which are generally controllable and specific. Chemical stimuli include pH and chemical reagents.

#### 3.2.1 pH

pH‐sensitive removal relies on altering the pH of the surrounding environment to trigger the dissolution of sacrificial materials. By adjusting the acidity or alkalinity, the process can be finely controlled, allowing precise manipulation of the removal process. However, significant shifts in pH can introduce cytotoxicity, particularly in biological systems where even slight changes in pH can harm cells or destabilize the surrounding matrix. Furthermore, achieving uniform pH changes across larger structures can be difficult, potentially leading to incomplete material removal. This limitation is especially critical in larger and more complex 3D constructs, where heterogeneous tissue architectures can lead to uneven diffusion and local pH gradients. These gradients not only reduce the efficiency of sacrificial material removal but also induce localized cytotoxicity. In order to address this problem, strategies such as localized pH modulation and controlled microfluidic delivery may be needed to ensure sufficient channel structural integrity, but they can complicate the entire procedure. Consequently, accurate regulation is essential to keep the pH within safe limits for the intended applications.

Shellac is a natural polymer resin secreted by the lac insect,^[^ ^124^ ^]^ which demonstrates pH‐responsive behavior, transitioning between insoluble and soluble states based on the surrounding pH. At acidic conditions (pH < 7), shellac remains insoluble and forms durable filaments, while under alkaline conditions (pH > 7), it ionizes and dissolves in aqueous media.^[^ ^125^ ^]^ This pH‐dependent solubility enables shellac to be removed by adjusting the pH of the system to alkaline conditions, making it suitable as a sacrificial material where controlled removal is required.^[^ ^90^ ^]^ Alternatively, an alkali‐soluble photopolymer, composed of *N*,*N*‐dimethylacrylamide (DMA), methacrylic acid (MA), methacrylic anhydride (MAA), and PVP, can be removed at alkaline conditions by adding NaOH.^[^ ^94^ ^]^

#### 3.2.2 Chemical Reagents

Chemical reagent‐based removal employs specific chemicals to break down sacrificial materials. While chemical reagents offer rapid and efficient material removal, their harsh nature can pose risks to surrounding structures, especially in sensitive biological environments where cytotoxicity is a concern. Thorough neutralization and washing steps are often required after the removal to eliminate residual chemicals, which also increases the complexity of the procedure. As a result, this method is best suited for non‐biological applications.

Alginate is a naturally derived polysaccharide from brown seaweed, known for its biocompatibility and capacity to form hydrogels via ionic cross‐linking.^[^ ^126^ ^]^ Gelation occurs when divalent cations, such as calcium ion (Ca^2^⁺) and barium ion (Ba^2+^), bind to the guluronic acid (G‐block) regions of the polymer, creating a stable 3D network.^[^ ^127^ ^]^ The network can be broken by introducing chelating agents such as ethylene diamine tetraacetic acid (EDTA) or sodium citrate, which leads to the removal of alginate hydrogels.^[^ ^96^ ^]^ EDTA and sodium citrate exhibit low cytotoxicity at moderate concentrations but may disrupt cell membranes and calcium homeostasis at high doses.^[^ ^128^, ^129^ ^]^

Beyond natural molecules, synthetic molecules are also employed to prepare sacrificial materials. The polyelectrolyte complex (PEC) is a complex of polyions with oppositely charged.^[^ ^130^ ^]^ The PEC formed by poly(diallyldimethylammonium chloride) and poly(sodium 4‐styrenesulfonate) provides tunable viscoelasticity and mechanical stability for sacrificial templates in tissue engineering, which can be removed by rapid dissolution in a potassium bromide aqueous solution.^[^ ^99^ ^]^ While potassium bromide shows minimal acute cytotoxicity, prolonged exposure has been associated with neurological effects due to bromism.^[^ ^131^ ^]^ Supramolecular interactions are introduced to fabricate sacrificial hydrogels. Dual‐component hydrogel formed with adamantane‐modified hyaluronic acid (Ad‐HA) and β‐cyclodextrin modified hyaluronic acid (CD‐HA) exhibits great shear‐thinning and self‐healing properties by host‐guest interaction. This hydrogel can be removed by introducing soluble β‐cyclodextrin to disrupt the host‐guest interaction.^[^ ^100^ ^]^ β‐cyclodextrin is usually biocompatible, but high concentrations may induce renal toxicity.^[^ ^132^ ^]^

In addition, a synthetic self‐healing hydrogel forms through the reversible crosslinking of polyethylene glycol‐diacrylate (PEGDA), dithiothreitol, borax, and branched polyethylenimine via thiol‐ene Michael addition, hydrogen bonds, and boronate ester bonds. This hydrogel is sensitive to glucose, which can disrupt the boronate ester bonds, breaking the hydrogel network and liquefying the hydrogel.^[^ ^102^ ^]^ Although essential for cellular metabolism, supraphysiological glucose levels can trigger oxidative stress and glycation, potentially leading to endothelial and neuronal cell dysfunctions.^[^ ^133^ ^]^ A thioester‐based elastomer is formed via the photoinitiated thiol‐ene ‘click’ reaction, where three‐arm PEG thiol and PEG thioester norbornene are crosslinked under ultraviolet (UV) light. It is selectively degraded via thioester exchange with 2‐mercaptoethanol, enabling rapid dissolution.^[^ ^103^ ^]^ 2‐Mercaptoethanol is highly cytotoxic, with strong reducing activity that disrupts protein disulfide bonds and impairs cellular viability, particularly affecting the liver and respiratory systems upon exposure.^[^ ^134^ ^]^

### 3.3 Biologically Triggered Sacrificial Materials

Biologically triggered sacrificial materials are defined as those designed to be selectively degraded or removed through biological stimuli, in which enzymatic degradation serves as the most typical approach. These methods are characterized by their high specificities and mild conditions, making them particularly suitable for applications requiring cytocompatibility and minimal disruption. Enzyme‐based removal utilizes specific enzymes that target and degrade sacrificial materials in a highly controlled and selective manner. The greatest advantage of this method is its specificity, as enzymes only act on the specific materials without affecting the surrounding structure. In addition, it is particularly beneficial in biological contexts, where the mild conditions required for enzyme activity help preserve cell viability and maintain structural integrity. Despite that, the enzymes are usually expensive, and the enzymatic process tends to be slow. Most of the aforementioned triggers typically enable the removal of sacrificial materials from several minutes to hours. In contrast, enzymatic degradation relies on the diffusion of enzymes, the density of recognition sites, and the structures of materials, often requiring several hours to days, especially in 3D hydrogels and cell‐laden environments where the diffusion is restricted. To achieve the desired degradation time, it is important to carefully select the appropriate enzyme, adjust its concentration, and ensure that the process is conducted under optimal temperature and pH conditions for maximal enzyme activity. In cases where enzymatic degradation still fails to meet the requirements, advanced strategies, such as protein engineering or synthetic biology, can be considered to design and modify enzymes to reach desired activities. Nevertheless, these approaches are often labor‐intensive and technically complex, which limits their practical applications in typical biofabrication processes.

Hyaluronic acid (HA) is a naturally occurring glycosaminoglycan composed of repeating units of *N*‐acetylglucosamine and glucuronic acid, which endow it with distinctive viscoelastic and bioactive properties.^[^ ^135^, ^136^ ^]^ It forms hydrogels through covalent crosslinking, ionic interactions, or hydrogen bonding. These hydrogels can be enzymatically degraded by hyaluronidase, which cleaves glycosidic bonds, allowing for precise removal and making HA highly suitable for applications requiring biodegradability and dynamic tissue remodeling.^[^ ^104^ ^]^

Deoxyribonucleic acids (DNAs) as a biopolymer with clear structure,^[^ ^137^ ^]^ good programmability,^[^ ^138^ ^]^ and biocompatibility,^[^ ^139^, ^140^, ^141^, ^142^ ^]^ serve as an ideal material type for hydrogel preparation,^[^ ^143^ ^]^ DNA hydrogels^[^ ^144^ ^]^ are typically formed through two primary mechanisms: chemical and physical crosslinking. Chemical cross‐linking^[^ ^145^ ^]^ utilizes DNA ligases to covalently connect DNA building blocks, while physical crosslinking^[^ ^146^ ^]^ relies on the self‐assembly of sticky ends, resulting in dynamic networks with permeability, lubrication, shear‐thinning, and self‐healing properties.^[^ ^147^ ^]^ DNA hydrogels can be degraded by enzymes under physiological conditions. Exonucleases degrade DNA in a nonspecific manner by progressively cleaving nucleotides from the ends of DNA strands, whereas restriction endonucleases recognize and cut at specific sites, allowing for controlled but relatively slow degradation.^[^ ^41^ ^]^ The enzymatic degradability of DNA makes it a suitable candidate for sacrificial materials.

Overall, although a variety of sacrificial materials based on physical, chemical, and biological removal methods have been developed, most remain limited in their ability to integrate high biocompatibility, capillary‐level fabrication, and rapid removal under physiological conditions. Bridging this gap calls for the exploration of unconventional and multifunctional materials that can meet the escalating demands of vascular biofabrication.

## 4 Surrounding Materials

**Figure 5:**
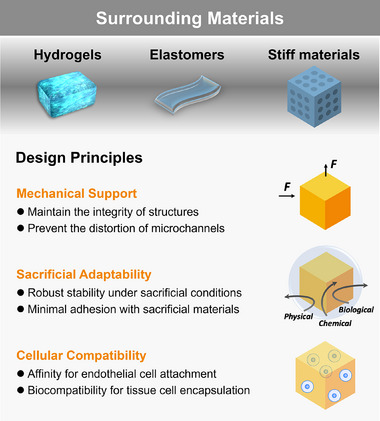
Classification and design principles of surrounding materials in sacrificial biofabrication.

The fabrication of vascularized constructs using the sacrificial biofabrication depends on more than just the sacrificial materials, it also requires choosing the compatible surrounding materials, which play a big role in keeping the structure integrity, making removal efficiency, and ensuring biological functionality. In this section, the classification of surrounding materials is briefly introduced, and the principles guiding surrounding material designs are discussed (**Figure** **5**).

### 4.1 Classification

In the sacrificial biofabrication, surrounding materials can be classified based on their physical state. Among them, hydrogels are the most commonly used due to their structural similarity to the extracellular matrix (ECM) and superior biocompatibility. In addition to hydrogels, elastomers, and stiff materials are also utilized as surrounding materials. The following sections will discuss these three categories in detail.

#### 4.1.1 Hydrogels

Hydrogels are 3D, hydrophilic networks that can retain large amounts of water while maintaining their structural integrity.^[^ ^148^, ^149^ ^]^ This unique property makes them highly similar to the natural ECM, providing a suitable environment for cell growth, proliferation, and differentiation.^[^ ^150^ ^]^ Commonly used materials in hydrogel fabrication include collagen,^[^ ^151^ ^]^ gelatin,^[^ ^105^, ^106^ ^]^ gelatin methacryloyl (GelMA),^[^ ^152^ ^]^ fibrin,^[^ ^153^ ^]^ agarose,^[^ ^43^ ^]^ Matrigel,^[^ ^154^ ^]^ alginate,^[^ ^155^ ^]^ PEG,^[^ ^156^ ^]^ PVA,^[^ ^111^ ^]^ and Pluronic F‐127.^[^ ^107^ ^]^

Based on the origin, hydrogels are broadly classified into natural hydrogels and synthetic hydrogels. Natural hydrogels are derived from plant or animal sources, giving them favorable biocompatibility and bioactivities.^[^ ^157^ ^]^ These materials inherently possess biofunctional motifs that facilitate cell adhesion. However, their structural complexity and compositional variability present challenges in reproducibility and precise modulation of mechanical properties. Conversely, synthetic hydrogels, such as PEG, PVA, and Pluronic F‐127, have well‐defined chemical structures, allowing for precise control over their mechanical and chemical properties, including stiffness, degradation rate, and functionalization potential.^[^ ^158^ ^]^ Yet, their bioactivities are generally lower than those of natural hydrogels.

Based on the cross‐linking mechanism, hydrogels can be categorized into physically cross‐linked and chemically cross‐linked hydrogels. Physically crosslinked hydrogels are formed through non‐covalent interactions such as hydrogen bonding, ionic interactions, or hydrophobic forces, resulting in dynamic and reversible networks, which endow physically crosslinked hydrogels with distinctive properties such as shear‐thinning, self‐healing, and shape memory.^[^ ^159^ ^]^ These properties make them advantageous for applications requiring dynamic remodeling or injectable formulations. In contrast, chemically crosslinked hydrogels are stabilized by covalent bonds, leading to more robust and permanent networks.^[^ ^160^ ^]^ The stable chemical bonds provide enhanced mechanical strength and structural integrity, rendering them less prone to swelling in culture media. Although they offer superior mechanical stability, the chemical cross‐linking processes may introduce cytotoxic residue and influence biocompatibility.

#### 4.1.2 Elastomers

Elastomers are flexible materials characterized by their excellent mechanical properties, including high elasticity, resilience, and durability.^[^ ^161^ ^]^ They are particularly useful in applications requiring stretchable scaffolds. Among the widely used elastomers, PDMS is popular due to its mature fabrication methods, ease of preparation, optical transparency, and favorable mechanical properties. However, its inherent hydrophobicity may require surface modification to enhance cell adhesion and compatibility.^[^ ^162^, ^163^ ^]^ Polyurethane (PU) is also extensively employed owing to its broad chemical variance, which allows for precise control over degradation rates and mechanical characteristics.

#### 4.1.3 Stiff Materials

Stiff materials are typically formed by synthetic polymers such as PLA, poly(lactic‐co‐glycolic acid) (PLGA), and PCL.^[^ ^164^ ^]^ These materials are commonly fabricated into scaffolds using 3D printing or spun into fibrous membranes using electrospinning technology.^[^ ^165^ ^]^ As the surrounding materials, they are typically first dissolved in organic solvents. The fabricated sacrificial templates are then immersed in the solution, leading to the formation of a liquid layer on the templates. Upon solvent evaporation, the liquid layer solidifies, effectively embedding the sacrificial templates within the surrounding materials. Subsequent removal of the sacrificial materials results in the formation of hollow channels within the surrounding materials.^[^ ^74^ ^]^ Stiff materials offer robust mechanical support and tailored degradation rates, making them suitable for long‐term structural applications, e.g., cardiac patch. However, their relatively low biocompatibility compared to hydrogels and elastomers may necessitate surface modifications or coatings to improve cell adhesion and bioactivity.

### 4.2 Design Principles

Generally, surrounding materials should meet these three requirements: mechanical support to preserve vascular architecture, sacrificial adaptability to withstand removal processes, and cellular compatibility to ensure cell growth, proliferation, and differentiation.

#### 4.2.1 Mechanical Support

The selection of surrounding materials is driven by their interactions with sacrificial templates during fabrication. The surrounding materials must provide sufficient mechanical strength^[^ ^166^ ^]^ to maintain the structural integrity of embedded sacrificial templates throughout the fabrication and subsequent processing stages. This strength is essential to prevent premature deformation, collapse, or displacement of the sacrificial templates, which could compromise the accuracy and functionality of the microchannels designed to mimic vascular networks. Additionally, another critical consideration is the swelling behavior of the surrounding materials in aqueous environments^[^ ^167^ ^]^ Since most tissue engineering and regenerative medicine applications involve hydrophilic or aqueous conditions, it is vital to carefully regulate the degree of swelling to prevent any obstruction or distortion of the microchannels. Uncontrolled swelling could lead to narrowing or blockage of channels, hindering the accurate replication of vascular networks.

#### 4.2.2 Sacrificial Adaptability

Surrounding materials must be compatible with the removal methods of sacrificial materials, as different strategies require specific chemical, mechanical, or thermal properties accordingly. Ideal surrounding materials should provide robust mechanics while remaining stable under the conditions required for sacrificial material removal, thereby avoiding undesired structural collapse, swelling, or degradation. Additionally, interfacial compatibility between the surrounding materials and sacrificial materials is crucial, ensuring easy separation during removal while maintaining channel fidelity. Specifically, the adhesion between the surrounding materials and sacrificial materials must be optimized to be weak enough to enable efficient removal without disrupting the vascular structure.^[^ ^168^ ^]^

Strategically pairing the surrounding materials with the removal methods ensures both high‐fidelity vascular architecture and efficient template removal. For example, to remove dissolvable templates such as PVA using a solvent‐based method, the surrounding materials should be resistant to solvent‐induced swelling or weakening. In contrast, thermosensitive sacrificial materials like Pluronic F‐127 liquefy at low temperatures (≈4 °C), making it essential for the surrounding materials to maintain their mechanical stability without undergoing temperature‐induced phase transitions. For chelation‐based removal of alginate templates using EDTA or sodium citrate, the surrounding materials must be resistant to Ca^2+^‐depletion to prevent destabilization. Additionally, for enzymatic degradation, the surrounding materials should not be susceptible to enzymatic action to avoid unintended breakdown.

#### 4.2.3 Cellular Compatibility

Beyond structural and chemical considerations, cellular compatibility is a key factor in surrounding material selection.^[^ ^169^ ^]^ Since engineered vasculature is designed to support endothelialization and subsequent vascular maturation, the surrounding materials must provide environments that facilitate cell adhesion, growth, and proliferation.^[^ ^170^ ^]^ Natural polymers such as collagen and gelatin are frequently used due to their biomimetic properties, which promote endothelial cell attachment and migration. Hybrid materials that combine synthetic polymers with bioactive components have also been explored to enhance cellular compatibility. These materials allow for controlled degradation, enabling dynamic remodeling of the engineered vasculature as cells proliferate and deposit their own ECM. Meanwhile, surrounding materials must possess permeability to encapsulate tissue cells, thereby fabricating vascularized tissues for disease models and regenerative medicine. Achieving a balance between mechanical integrity and biological functionality is essential for ensuring long‐term vascular integration.

With the advancement of tissue engineering, numerous biomaterials have been reported, all of which could potentially be used as surrounding materials for vascular biofabrication. Nonetheless, most of them are not able to replicate the multilayered and multicellular architecture of native vasculature. Achieving this fidelity will require advanced strategies for the fabrication of layer‐by‐layer structures with distinct cell types positioned in defined layers.

## 5 Technologies

Beyond selecting the appropriate sacrificial materials, another crucial aspect of the sacrificial biofabrication is determining how to shape these materials into vascular patterns. The formation of well‐defined vascular networks is the foundation for creating functional tissues.

**Figure 6:**
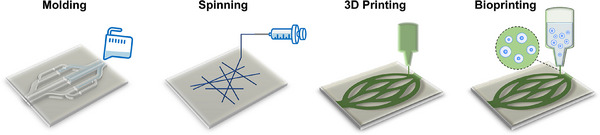
Scheme of different technologies for shaping sacrificial materials into vascular patterns, including molding, spinning, 3D printing, and bioprinting.

In this section, we focus on the technologies for shaping sacrificial materials into vascular structures. The four most common methods include molding, spinning, as well as 3D printing and bioprinting (**Figure** **6**). Each of these approaches offers distinct advantages and limitations depending on the specific application. In the following, representative examples of these methods are explored, and the evaluation of their respective strengths and drawbacks is provided (Table 2). Since the resolution of vasculature is critical for mimicking the dimensions of human capillaries, the smallest diameter of acellular vascular structure (D_v_) and the smallest diameter of endothelialized vasculature (D_e_) for each example are also listed.

### 5.1 Molding

**Figure 7:**
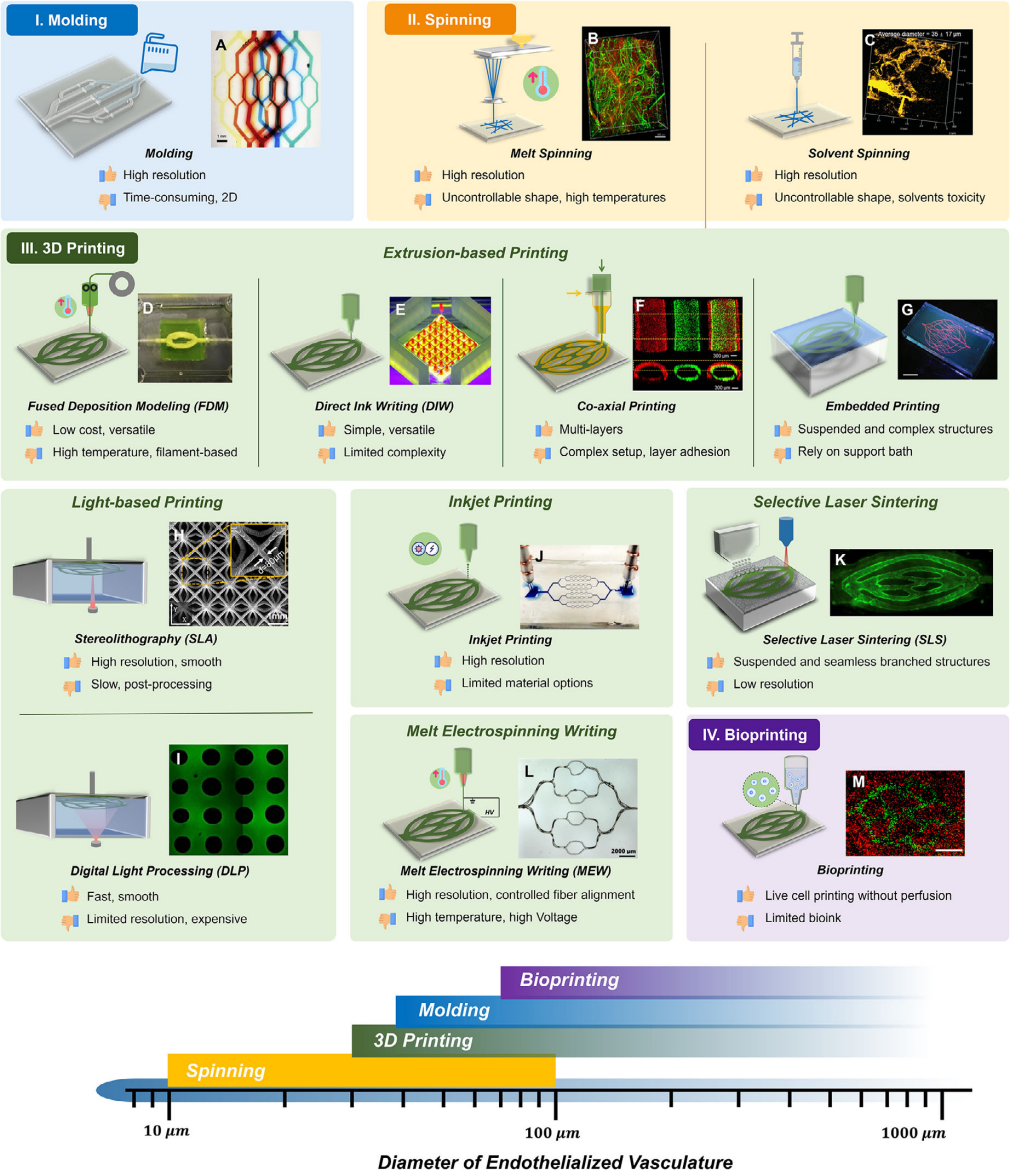
Illustrations, examples, and diameters of molding, spinning, 3D printing, and bioprinting technologies used in sacrificial biofabrication for vascularization. A) Image of microfluidic gel perfused with fluorescent microspheres. Reproduced with permission.^[^ ^69^ ^]^ Copyright 2015, Elsevier. B) Microchannels in collagen hydrogels perfused with green fluorescent microbeads. Reproduced with permission.^[^ ^93^ ^]^ Copyright 2017, Wiley‐VCH. C) Confocal microscopic image of microvascular networks with gelatin hydrogels. Reproduced with permission.^[^ ^61^ ^]^ Copyright 2016, Wiley‐VCH. D) Photograph of the printed PVA branch in the gelatin surrounding the hydrogel construct. Reproduced with permission.^[^ ^71^ ^]^ Copyright 2018, Elsevier. E) Image of sacrificial inks (red) and cell inks (green) printed on the chip. Reproduced with permission.^[^ ^173^ ^]^ Copyright 2016, National Academy of Sciences. F) Fluorescence images of TEBVs with HUVECs (green) and HASMCs (red). Reproduced with permission.^[^ ^174^ ^]^ Copyright 2020, IOP Publishing. G) Microvascular networks with photo‐crosslinked Pluronic F‐127 gel reservoir. Reproduced with permission.^[^ ^55^ ^]^ Copyright 2011, Wiley‐VCH. H) SEM image of a printed lattice structure. Reproduced with permission.^[^ ^95^ ^]^ Copyright 2021, Elsevier. I) Image of the Matrigel patterned with printed crypt structures. Reproduced with permission.^[^ ^103^ ^]^ Copyright 2021, IOP Publishing. J) Microvascular networks in GelMA hydrogel perfused with blue dye. Reproduced with permission.^[^ ^56^ ^]^ Copyright 2021, IOP Publishing. K) Green fluorescent protein‐HUVECs adhered to vascular channels within GelMA hydrogels. Reproduced with permission.^[^ ^176^ ^]^ Copyright 2020, Springer Nature. L) Image of a branched structure fabricated by print‐and‐fuse strategy. Reproduced with permission.^[^ ^64^ ^]^ Copyright 2022, Wiley‐VCH. M) Fluorescent image of vascularized liver tissue construct. Reproduced with permission.^[^ ^41^ ^]^ Copyright 2024, Wiley‐VCH.

Molding signifies casting sacrificial materials into pre‐designed molds that define the vascular shapes. Once solidified, the sacrificial materials are encased within surrounding materials and then removed, leaving hollow channels for vascularization (**Figure** **7**). In 2007, Tien and co‐workers first fabricated the vascular network through the sacrificial biofabrication by employing gelatin as a sacrificial material.^[^ ^38^ ^]^ In this study, gelatin was micromolded into a precise mesh and encapsulated within a hydrogel (collagen/fibrinogen/matrigel), and then removed through heating and flushing to leave interconnected microchannels. The channel accurately preserved the structural details of the original gelatin mesh with a fine resolution of 6 µm, while increasing to ≈50 µm upon seeding with endothelial cells. Apart from the network formation, effective incorporation of macromolecules and particles into the channels, along with their subsequent diffusion from the channels into the surrounding gel matrix, was also demonstrated.

In a later work, sodium alginate was used as a sacrificial material to engineer 3D microfluidic vascular structures in hydrogels.^[^ ^96^ ^]^ Alginate solution was rapidly crosslinked via Ca^2+^ to form the hydrogel in patterned PDMS microfluidic channels and completely encapsulated in another hydrogel (gelatin/collagen/agarose). Dissolution of the sacrificial alginate templates with EDTA solution led to the formation of interconnected vascular networks with defined dimensions and morphology ranging from 20 to 500 µm, which provided a substrate for the proliferation of human umbilical vein endothelial cells (HUVECs), enabling the formation of vascular lumens. Furthermore, macromolecules (20 and 70 kD dextran) were used to assess the permeability of the endothelial layer. Although the permeability of their vascular model was still much higher than native blood vessels, the results demonstrated the development of a confluent endothelial monolayer, indicating the potential of sacrificial biofabrication to achieve the actual endothelial barrier functions.

In addition to gelatin and sodium alginate, PVA could also be used as the sacrificial material.^[^ ^69^ ^]^ In this work, PVA templates were cast and shaped in the mold, encapsulated within hydrogel matrices (2‐hydroxyethyl methacrylate (HEMA)/GelMA/agarose), and later dissolved in aqueous media to reveal perfusable channels (Figure 7A). The resulting vascular network was as narrow as 100 µm. Cell seeding, mass transportation, and perfusion stability were also tested in the following experiments, exhibiting that the vascular networks could prevent necrosis within the core of thick tissue constructs, which expands the dimensions of current engineered tissues to clinical applications.

The aforementioned studies employ artificially engineered templates to mold sacrificial materials. Although it is possible to fabricate specific vascular shapes, the complexity remains far inferior to that of native vasculatures. Yoo and co‐workers described a novel method for fabricating biomimetic vascular scaffolds using renal tissue as the template.^[^ ^84^ ^]^ In this report, sacrificial PCL was perfused into renal tissue to replicate native vascular structures. Afterwards, the tissue was digested and the PCL template was obtained. This PCL template was coated with collagen hydrogel and then dissolved with acetone, resulting in a hollow collagen scaffold with the same architecture as normal renal tissue. The scaffold channels achieved a resolution close to native vasculatures, which could be seeded with cells, perfused, and endothelialized. This method offered a straightforward, cost‐effective way to create biomimetic, tissue‐specific vascular scaffolds suitable for broad applications in regenerative medicine. Beyond their intricate geometries, the multilayered architecture is also a key characteristic of blood vessels. Therefore, in order to get a biomimetic, multilayered vascular construct in vitro, a diffusion‐induced gelation method was combined with sacrificial biofabrication to fabricate multilayer tubular structures.^[^ ^171^ ^]^ In this study, Ca^2+^loaded gelatin hydrogel was cast into a mold to achieve a defined shape, serving as a sacrificial material. The gelatin core was subsequently immersed in alginate solution, allowing the crosslinking of the surrounding alginate layer by the diffusion of encapsulated Ca^2+^. After successive immersion cycles, multiple layers of alginate hydrogel were deposited around the gelatin core. Finally, the gelatin core was removed thermally, resulting in the formation of multilayered tubular alginate hydrogels. Results showed that as many as seven alginate hydrogel layers with clear boundaries could be prepared in 2–3 min. The diameter of the gelatin rod was 1‐5 mm and the resolution of the layer dimension was 25 µm. Different types of cells, such as HUVECs, smooth muscle cells (SMCs), and fibroblasts, were successfully encapsulated into distinct layers of the alginate hydrogel with viability exceeding 90%, highlighting the potential of this method to replicate the native multilayered structures of blood vessels.

Recently, Chen and co‐workers presented a Ga‐based engineered sacrificial capillary pump for evacuation (ESCAPE) to fabricate hierarchical vascular networks within soft hydrogels.^[^ ^91^ ^]^ Ga was employed as the sacrificial material and cast into predefined structures using a PDMS mold. The Ga template was then embedded in collagen, agarose, or fibrin matrices at room temperature and subsequently heated to 32 °C to melt into a liquid state. Its removal was driven by capillary forces generated through the creation of geometric asymmetry and the dissolution of the native oxide layer. The resulting vascular structures achieved a resolution of 30 µm, while after endothelial cell seeding, the vessel diameter was 39 µm. The engineered vascular networks were evaluated in perfusion studies, demonstrating effective fluid transport within biomimetic tissue constructs. This approach offers a scalable, high‐fidelity method for fabricating multiscalar vascular architectures, spanning approximately from 300‐µm arterioles down to 30‐µm microvasculatures, which addresses the longstanding challenges in sacrificial biofabrication.

The molding technique allows precise control over geometry, enabling detailed vascular networks. Nonetheless, the approach is often limited by time‐consuming manual processes because each mold requires customization and careful handling to preserve structural integrity. At the same time, this technique is generally constrained to fabricating 2D structures, lacking the capacity to replicate the intricate 3D architecture of actual vasculatures.

### 5.2 Spinning

The spinning technology is applied to process sacrificial materials into fibrous structures, rendering it ideal to create extremely fine channels for vascular applications.^[^ ^180^ ^]^ This technology encompasses two primary approaches based on material states: melt spinning and solution spinning (Figure 7).

#### 5.2.1 Melt‐Spinning

In melt‐spinning, sacrificial materials are heated to a molten state and extruded into fine filaments, which solidify upon cooling.^[^ ^181^ ^]^ Bellan and co‐workers first used melt spinning to construct 3D vascular networks using sugar as sacrificial material.^[^ ^73^ ^]^ Melt‐spun sugar fibers, produced with a modified cotton candy machine, were used to create channels resembling natural capillaries in size and density. The sugar structures were embedded in PDMS and dissolved afterwards in aqueous media and ethanol, leaving behind hollow microchannels. The networks had capillary‐scale resolution of ≈1–100 µm and could support physiologically relevant flow rates and pressures, which was verified by perfusion of fluorescent particles and blood. Compared with the molding strategy, this method offers significantly higher resolution but suffers from the limited shape controllability and the requirement of elevated temperatures. Lithography technology was also introduced in this system to pattern PDMS substrate.^[^ ^172^ ^]^ By combining lithography technology and sacrificial biofabrication, hierarchical vascular networks with large lithographically patterned channels and small sacrificial channels were prepared. This scalable and versatile approach seamlessly bridges macroscale and microscale fluidic system, with a resolution of 10 µm in channel diameter, which closely resembles the size of natural capillaries. Later, shellac was used as a sacrificial material to fabricate 3D vascular networks in gelatin hydrogel.^[^ ^92^ ^]^ Shellac was chosen over sugar due to its pH‐sensitive solubility, which enables easier and more controlled removal under mild conditions. PDMS was replaced with gelatin hydrogel for its superior biocompatibility, making it more suitable for cell encapsulation. Similarly, melt‐spun shellac fibers, generated using a modified cotton candy machine, were embedded within enzymatically crosslinked gelatin hydrogel. After removing shellac with an ammonium hydroxide solution, hollow channel networks were created in the gelatin substrate. The fabricated networks exhibited an average channel diameter of 17 µm with interchannel spacing optimized for nutrient diffusion.

Spector and co‐workers presented a refined method to fabricate hierarchical vascular networks in biocompatible tissue‐engineered constructs using Pluronic F‐127 and melt‐spun shellac fibers as sacrificial materials.^[^ ^93^ ^]^ Macrochannels were established with Pluronic F‐127, while shellac fibers were melt‐spun to generate a dense network of microchannels. These fibers were embedded within type I collagen hydrogels, and the sacrificial materials were sequentially dissolved using ethanol and PBS buffer to leave behind hollow channels. The resulting microchannels ranged from 10–90 µm in diameter, mimicking natural capillary dimensions (Figure 7B). Endothelial and smooth muscle cells were seeded into the constructs, forming hierarchical vascular networks with cluster of differentiation CD31 expressed endothelial linings in small channels (<25 µm) and smooth muscle layers in larger channels (>25 µm). Flow continuity and robust cellular adhesion were also confirmed by functional tests.

The melting‐spinning technology benefits from high resolution that could reach the nanoscale, but the shape and size of the microchannels are generally uncontrollable. Simultaneously, the elevated temperatures in melt spinning impose constraints on material selection and pose challenges for biomedical applications.

#### 5.2.2 Solution Spinning

In contrast to melt‐spinning, solution spinning is a fiber fabrication process where a solution of sacrificial material is extruded and the solvent is subsequently removed via evaporation or coagulation in a nonsolvent bath to solidify fibers.^[^ ^182^ ^]^ Thermoresponsive PNIPAM was employed as a sacrificial template to construct 3D microvascular networks within gelatin hydrogels through solvent spinning.^[^ ^61^ ^]^ PNIPAM fibers, with diameters ranging from 3 to 55 µm, were solvent‐spun and encapsulated into gelatin hydrogels. Due to its thermoresponsive property, PNIPAM remains stable during the gelatin crosslinking process and can be subsequently removed under mild conditions at room temperature. The resulting channels had an average diameter of 35 µm, with the resolution influenced by fiber swelling during the embedding process (Figure 7C). Fibroblasts were embedded into gelatin hydrogels, and it was demonstrated that perfusion of cell medium through the microchannel networks significantly improved fibroblast survival and function, with over 96% cell viability in perfused constructs on days 2 and 7. PVA was also used as a sacrificial material to fabricate mechanically anisotropic poly(glycerol sebacate) (PGS) membranes for vascular tissue engineering.^[^ ^70^ ^]^ Electrospun PVA fibers were aligned on a rotating drum to create fibrous membranes, which were partially immersed in PGS prepolymer and cured under vacuum. The sacrificial PVA fibers were then removed via water dissolution, resulting in aligned cylindrical pores within the PGS membrane. The vascular networks displayed pore sizes consistent with fiber diameters of 189 nm and structural anisotropy, as verified by scanning electron microscope (SEM) and atomic force microscopy (AFM). Tubular scaffolds constructed from these membranes exhibited compliance properties determined by the pitch angle of their double‐helical structure, mimicking arterial mechanics. Cytocompatibility assays using human umbilical artery smooth muscle cells (HUASMCs) confirmed the absence of cytotoxicity.

In a later study, multiscale vascular networks were prepared within a gelatin matrix by integrating 3D printing and electrospinning technologies.^[^ ^85^ ^]^ Pluronic F‐127 and PCL were utilized as sacrificial materials to create macrochannels and microchannels, respectively. The sacrificial materials were removed by cooling for Pluronic F‐127 and dissolution using dichloromethane for PCL. The diameters of macrochannels ranged from 400–600 µm, while microchannels were between 2‐20 µm. HUVECs were seeded in the macrochannels and showed an initial viability of 88.5%, which remained above 85% after 7 days.

The solution spinning technology similarly presents the benefit of high resolution, yet is hindered by limitations in shape control. In addition, solvent spinning often raises safety concerns due to the toxicity of solvents and is challenging to scale up production.

### 5.3 3D Printing

3D printing is a versatile additive manufacturing technology that often builds objects point‐by‐point or layer‐by‐layer.^[^ ^183^ ^]^ By precisely depositing materials according to digital models, it enables the creation of intricate, customized structures that would be difficult to produce using traditional methods.^[^ ^184^ ^]^ Widely adopted across fields such as aerospace, healthcare, and automotive engineering, 3D printing serves applications ranging from rapid prototyping to the manufacture of functional components. In the sacrificial biofabrication, 3D printing stands as the preferred technology for processing sacrificial materials, valued for its capability to achieve meticulously designed geometries. Key methods for 3D printing sacrificial materials include extrusion‐based printing, light‐based printing, inkjet printing, selective laser sintering (SLS), and melt electrospinning writing (MEW) (Figure 7D–L), with extrusion‐based printing being the predominant choice due to its versatility and reliability.

#### 5.3.1 Extrusion‐Based Printing

Extrusion‐based printing^[^ ^185^ ^]^ operates by extruding materials through a nozzle to build structures layer‐by‐layer. This technology enables precise control over material deposition, making it highly versatile and suitable for various applications. Beyond traditional fused deposition modeling (FDM) and direct ink writing (DIW), advanced technologies have emerged through enhancements in printing mechanisms and methodologies. These contain co‐axial printing and embedded printing. Such advancements broaden the capabilities and intricacies achievable through extrusion‐based printing.

##### FDM

FDM processes sacrificial materials by heating them to a molten state and extruding them through a nozzle, where they are deposited layer by layer to form a predefined 3D structure.^[^ ^186^ ^]^ In 2012, Chen and co‐workers first used 3D printing technology to process sacrificial material in the sacrificial biofabrication.^[^ ^76^ ^]^ Carbohydrate glass, used as a sacrificial material, was printed into rigid 3D lattices with multiscale architecture, encapsulated in ECM‐based hydrogels (agarose/alginate/PEG/fibrin/Matrigel), and subsequently dissolved in cell culture media to create hollow cylindrical channels. The fabricated vascular channels had diameters ranging from 200 µm to 1 mm. HUVECs were seeded into the channels and formed confluent layers within 24 h. Channels were confirmed to be able to support laminar and pulsatile flow under physiological pressures, which effectively sustained the metabolic activity and functionality of primary hepatocytes in ECM constructs by perfusion.

PVA was used as a sacrificial material to 3D print vascular networks in thick (1 cm) and densely populated (10^7^ cells/mL) tissue constructs.^[^ ^71^ ^]^ At first, PVA branched structures were fabricated by co‐printing with PLA as surrounding structures. After dissolving the PLA with chloroform, gelatin‐based hydrogels were cast over the PVA structures and crosslinked using transglutaminase (Figure 7D). PVA template was subsequently removed via perfusion with aqueous media, yielding hollow vascular channels with a resolution of ≈1.3 mm. The constructs supported perfusion flow rates of up to 2 mLmin^−1^ under physiological pressures. Hepatocellular carcinoma cells encapsulated in gelatin hydrogels exhibited high viability (>90%) and formed functional spheroids over 14 days of perfusion.

The primary advantage of the FDM technology is its cost‐effectiveness and versatility since it supports a diverse range of thermoplastic materials. However, the technology necessitates high temperatures for extrusion and filament‐based materials, as most commercial FDM printers rely on filament‐based feedstocks with a highly consistent diameter, typically around 1.75mm, which requires pre‐processing of raw materials before they can be used.

##### DIW

DIW refers to the direct extrusion of sacrificial materials through a nozzle onto the build platform, where it is layered sequentially to form a defined shape.^[^ ^187^ ^]^ Lewis and co‐workers utilized Pluronic F‐127 as a sacrificial material to 3D print microvascular networks within GelMA hydrogels.^[^ ^53^ ^]^ Pluronic F‐127 was printed into various structures from one‐dimensional (1D) to 3D, cast with GelMA hydrogels, and then removed by cooling the construct below 4 °C, leaving open channels in GelMA constructs with diameters ranging from ≈ 100 µm to 1 mm. HUVECs could maintain over 95% of viability and assemble into continuous layers after being injected into networks for 48 h. Human neonatal dermal fibroblasts and 10T1/2 fibroblasts were also encapsulated in the GelMA bioink and co‐printed with fugitive Pluronic F‐127 ink. After seeding with HUVECs, heterogeneous engineered tissue constructs with endothelialized channels were finally formed. Later, 3D printing of thick vascularized tissues with embedded vasculature and heterogeneous cell compositions was successfully fabricated.^[^ ^173^ ^]^ The previous approach relied on UV light to crosslink the GelMA matrix, which was constrained by the low penetration depth of UV light, thereby limiting the achievable tissue size. In this study, enzymatic crosslinking of the gelatin hydrogels was employed, enabling the fabrication of tissue constructs with dimensions up to 1 cm (Figure 7E). HUVECs were seeded into the channels, and other types of tissue cells were embedded in a gelatin matrix. With the elongation of perfusion time to 45 days, nutrient and oxygen delivery within thick tissue constructs supported long‐term cell viability and osteogenic differentiation.

The DIW technology is simple and versatile, accommodating a wide range of materials. Even so, as it relies on the point‐by‐point deposition principle, it cannot print suspended or complex structures. Realizing more advanced geometries necessitates further refinement and modifications to this approach.

##### Co‐Axial Printing

Co‐axial printing enables the simultaneous deposition of multiple materials through a co‐axial nozzle, allowing for the fabrication of intricate core‐shell structures.^[^ ^188^ ^]^ Cho and co‐workers employed an advanced 3D co‐axial printing technology to fabricate bioengineered blood vessels (BBVs) with precise structures and functions.^[^ ^54^ ^]^ Sacrificial Pluronic F‐127 ink was used as the core material and extruded through the inner nozzle, while a hybrid bioink composed of vascular‐tissue‐derived decellularized ECM (VdECM) and alginate was extruded through the outer nozzle to form the vessel walls. After thermal stabilization and the removal of Pluronic F‐127 by cooling, the resulting hollow channels had diameters ranging from 500 to 1500 µm, with a wall thickness of 50–200 µm. Endothelial progenitor cells seeded within the channels formed functional endothelial layers. The BBVs were loaded with therapeutic agents and demonstrated sustained drug release. In vivo studies demonstrated enhanced vascularization and significant limb regeneration in ischemic models. We employed a coaxial extrusion‐based biofabrication approach to construct perfusable conduits.^[^ ^72^ ^]^ The sacrificial material PVA served as the inner core, while the surrounding material, consisting of a gelatin/GelMA hydrogel, constituted the outer layer. Gelatin played a crucial role in initial thermo‐crosslinking, providing mechanical stability during extrusion, whereas GelMA underwent UV photocross‐linking, ensuring long‐term structural integrity. Gelatin was ultimately removed at 37 °C, enhancing porosity and facilitating cell infiltration. PVA was later washed out with warm PBS, leaving behind open and perfusable channels. Optimization of nozzle gauge combinations enabled the fabrication of the perfusable vascular structure with an inner diameter of 251 µm and an outer diameter of 610 µm. Notably, HUVECs were successfully seeded into the outer GelMA layer, demonstrating viability and endothelialization.

Bilayered tissue‐engineered blood vessels (TEBVs) were also formed using a co‐axial printing method that closely resembled the architecture of native blood vessels.^[^ ^174^ ^]^ Using a triple‐concentric nozzle, tubular constructs were designed with three distinct components: the inner core consisted of Pluronic F‐127 as sacrificial material, the middle layer was composed of collagen type I mixed with HUVECs, and the outer layer combined sodium alginate, collagen, and human aortic smooth muscle cells (HASMCs) to provide mechanical support. Following removal of Pluronic F‐127 core, bilayered hollow fibers with outer diameters of 1500‐1700 µm and inner diameters of 1300–1500 µm were prepared. The wall thickness was 90‐120 µm. The constructs maintained a cell viability of over 90% after 20 days of culture (Figure 7F). In addition, a significant proportion of HUVECs and HASMCs were oriented parallel and perpendicular to the TEBVs, respectively, mimicking the physiological alignment observed in vivo.

Although the co‐axial printing technology is especially useful for producing dual‐layered or hollow structures, making it ideal for replicating blood vessels with distinct inner and outer layers, the setup of this technology is relatively complex. Meanwhile, this method demands accurate coordination of different materials, sometimes presenting challenges in maintaining consistent layer adhesion throughout the process.

##### Embedded Printing

Embedded printing is an enabling 3D printing technology tailored for the meticulous fabrication of intricate structures.^[^ ^189^ ^]^ During the printing process, the sacrificial materials are directly extruded into other materials, which serve as support baths. After removing sacrificial materials, vascular channels can be formed within support baths. Lewis and co‐workers used embedded printing to fabricate 3D microvascular networks within a photopolymerizable hydrogel matrix.^[^ ^55^ ^]^ Pluronic F‐127 was used as a sacrificial material and 3D‐printed directly into a diacrylate‐functionalized Pluronic F‐127 gel reservoir. The hydrogel matrix was photocrosslinked with UV light, and the Pluronic F‐127 ink was subsequently removed under cooling conditions, generating hollow microchannels (Figure 7G). The resolution of the vascular networks reached the scale of capillaries and small blood vessels, which was 18 µm. Solute transport and flow dynamics were validated to demonstrate the potential for efficient nutrient and oxygen delivery.

Dual‐component hydrogels with modified HA were also used as sacrificial materials to prepare microvascular networks.^[^ ^100^ ^]^ The sacrificial ink was composed of adamantane‐modified HA (Ad‐HA) and β‐cyclodextrin‐modified HA (CD‐HA), forming shear‐thinning, self‐healing hydrogels by host‐guest interaction. The support hydrogel shared a similar composition, with Ad‐HA further modified with norbornene (AdNor‐HA) to enable covalent crosslinking via a thiol‐ene reaction under UV exposure. By precisely adjusting the properties of the sacrificial ink and the support hydrogel, the sacrificial ink was directly 3D‐printed into the support reservoir. After UV crosslinking of the support hydrogel, soluble β‐cyclodextrin was introduced to disrupt the host‐guest interaction, removing the sacrificial ink and leaving a hollow channel. The incorporation of adhesive peptides in the support hydrogel facilitated the formation of a confluent endothelial monolayer along various channel geometries, including straight, stenotic, and spiral designs. When protease‐degradable crosslinkers and angiogenic factor gradients were introduced, endothelial cells sprouted directionally into the support hydrogel, with enhanced sprouting at curved regions. These angiogenesis‐derived vessels are typically at the capillary level. As such, the printed vessels and new capillaries will form a hierarchical vascular network that is the most efficient system to perfuse blood into thick tissue. Another study used alginate as a sacrificial material to create microvascular networks by embedded printing.^[^ ^97^ ^]^ Alginate was directly printed into a support bath preloaded with Ca^2^⁺ (e.g., CaCl_2_) to enable immediate crosslinking into stable calcium‐alginate (Ca‐Alg) hydrogels. The support hydrogel, composed of materials such as agarose, gelatin, or GelMA, was solidified through thermal, enzymatic, or photopolymerization mechanisms. After that, vascular channels with a resolution of 200–300 µm were formed by removing Ca‐Alg hydrogels using sodium citrate. It has been demonstrated that HUVECs adhered to the channel walls after seeding, which formed confluent monolayers while maintaining the channel diameters.

Co‐axial printing^[^ ^190^ ^]^ was combined with embedded printing to fabricate tubular vascular structures.^[^ ^79^ ^]^ The sacrificial material, carbomer hydrogel, serves as the inner core in the coaxial 3D printer to form the lumen, while UV‐cross‐linkable bioelastomer prepolymer was used as the outer layer to form the vessel wall. These two materials were co‐printed into a carbomer hydrogel support bath, which provided mechanical support to prevent deformation of structures during the printing process. Then, the bioelastomer was photopolymerized under UV light to solidify the outer wall, and the sacrificial core was removed via PBS washing, forming tubular vascular structures. The minimum diameter of the microtube was 300 µm with wall thicknesses of 100–200 µm. Endothelial cells were successfully seeded within the lumen, and cardiac tissue was cultured around the vascular network, highlighting the potential for advanced applications in tissue engineering and regenerative medicine.

Sacrificial writing into functional tissue (SWIFT) is a new variation of embedded printing in which sacrificial materials are printed into the support baths containing live cells.^[^ ^47^ ^]^ Lewis and co‐workers employed the SWIFT technology to construct perfusable vascular networks in densely cellular organ building block (OBB) matrices. Gelatin was used as sacrificial ink and printed into a living matrix comprising embryoid bodies and ECM under cold conditions to maintain matrix fluidity. Afterward, the construct was warmed to 37 °C, solidifying the ECM and liquefying the gelatin, which was subsequently removed to form hollow vascular channels. The fabricated channels had varying diameters from 400 µm to 1 mm, depending on the printing parameters, and supported endothelial cell seeding to achieve functionalized lumens. As a proof of concept, perfusable cardiac tissues were fabricated that achieved synchronous beating after culturing for 7 days. In order to form biomimetic vascular networks with multiple layers, co‐axial printing was introduced in SWIFT technology, which was termed co‐SWIFT.^[^ ^175^ ^]^ The core material was sacrificial gelatin hydrogel, and the shell layer was composed of collagen, optionally laden with SMCs to replicate native vessel walls. Printing was performed at low temperatures (≈4 °C), followed by incubating at 37 °C to solidify the collagen shell and liquefy the gelatin core. The removal of the sacrificial core results in hollow, perfusable vascular channels with diameters between 125 µm and 1 mm. Endothelial cell seeding on the channels produced functional lumens with effective barrier functions and physiological morphology. Notably, co‐SWIFT‐derived cardiac tissues matured under perfusion, achieving synchronized contractions and exhibiting a cardio‐effective drug response in vitro.

The embedded printing technology allows for the creation of suspended and complex structures without gravity‐induced distortion that are difficult to achieve with conventional technologies, but the reliance on support baths would slow down the production and necessitate specific sacrificial materials that are compatible with support baths, thus slightly limiting versatility.

#### 5.3.2 Light‐Based Printing

Light‐based printing^[^ ^191^ ^]^ is a technology that uses light to create structures or patterns on a material with high precision. It works by selectively exposing photosensitive materials to specific wavelengths, triggering chemical reactions that solidify the materials. There are various types of light‐based printing, among which stereolithography (SLA) and digital light processing (DLP) are the predominant technologies employed in the sacrificial biofabrication.

##### SLA

SLA fabricates sacrificial materials by selectively curing a liquid photopolymer resin with a focused laser, inducing solidification to achieve a highly precise 3D structure.^[^ ^192^ ^]^ An alkali‐soluble photopolymer, composed of DMA, MA, MAA, and PVP, was used as the sacrificial material to fabricate porous structures.^[^ ^94^ ^]^ The sacrificial mold was created using SLA and filled with different biomaterials, such as PLGA, PCL, PLA, chitosan, alginate, and bone cement. Then the sacrificial mold was removed by NaOH. The resultant structure had a resolution of 50 µm for pores and 65 µm for struts, and the biocompatibility was confirmed by in vitro cytotoxicity assays. Later, this alkali‐soluble photopolymer was also used to fabricate porous structures in PEGDA surrounding materials with the resolution of 80 µm (Figure 7H).^[^ ^95^ ^]^

The SLA technology offers exceptional resolution and a smooth surface finish, making it particularly advantageous for applications requiring intricate details, such as biomedical models and precision engineering. However, its sequential point‐by‐point laser curing significantly limits printing speed. Additionally, post‐processing is necessary, including resin cleaning, support removal, and secondary UV curing, which adds complexity and additional time to the overall fabrication workflow.

##### DLP

DLP printing uses projected light patterns to selectively cure sacrificial materials layer by layer, enabling rapid and high‐resolution printing.^[^ ^192^ ^]^ DLP was employed to form 3D organoid structures using a sacrificial thioester‐based elastomer template.^[^ ^103^ ^]^ The sacrificial material comprises three‐arm PEG thiol and PEG thioester norbornene, which undergo thiol‐ene photopolymerization to form a degradable crosslinked network. After being printed using DLP, the sacrificial elastomer was put onto Matrigel or PEG hydrogels. Thioester exchange‐mediated degradation with 2‐mercaptoethanol enabled the removal of the sacrificial elastomer within 2 h, yielding structures within surrounding hydrogels with a resolution of 22 µm (Figure 7I).

The DLP technology is highly suitable for producing smooth surfaces and enables fast fabrication, as it solidifies resin layer by layer rather than the point‐by‐point mode used in SLA, thereby significantly reducing printing time. But its resolution is inherently constrained by the projector's pixel density, making it less precise than laser‐based systems. The cost of high‐resolution DLP projectors and specialized photopolymers is also sometimes prohibitive.

#### 5.3.3 Inkjet Printing

Inkjet printing is a droplet‐based deposition technology that employs thermal or piezoelectric actuation to eject precise volumes of ink through nozzles onto a substrate.^[^ ^193^ ^]^ This approach is well‐suited for sacrificial material deposition, particularly when high‐resolution patterning is required. Gelatin was used as a sacrificial material to create vessel models with three distinct layers through inkjet printing.^[^ ^48^ ^]^ Gelatin acted as the core of the vascular channel, while fibrin and collagen hydrogels acted as the support matrix. The gelatin core was printed at 5 °C, rapidly solidifying upon deposition, and later removed by warming to 37 °C. The resulting endothelialized vascular structures featured a diameter of 1 mm and a wall thickness of 425 µm. The constructs were dynamically cultured in bioreactors under physiological flow for up to three weeks. Pluronic F‐127 was also used as a sacrificial material to fabricate microvascular tissue constructs with hierarchical and branching networks by electrohydrodynamic (EHD) inkjet printing.^[^ ^56^ ^]^ Pluronic F‐127 was first printed at microscale precision to create channel templates and then cast with GelMA matrix. After photopolymerization of the GelMA, Pluronic F‐127 was removed at 4 °C, resulting in well‐defined, hollow vascular channels with a resolution of 30 µm (Figure 7J). When seeded with HUVECs, the channels developed confluent endothelial monolayers, producing functionalized endothelialized lumens. This approach further enabled the co‐culture of fibroblasts and endothelial cells to generate vascularized tissues that maintain high cell viability for up to 21 days.

A notable advantage of inkjet printing is its high resolution, enabling the fabrication of intricate microscale structures. However, the primary limitation lies in its compatibility with only low‐viscosity inks, which constrains the range of materials that can be used.

#### 5.3.4 SLS

SLS utilizes a laser to selectively fuse powdered sacrificial materials layer‐by‐layer according to the digitally defined 3D models.^[^ ^194^ ^]^ The powders are heated to a temperature just below their melting points, promoting particle fusion and forming precise structures. Miller and co‐workers described a method for fabricating vascular networks using sacrificial carbohydrate templates created through SLS.^[^ ^176^ ^]^ The templates, made from isomalt, were patterned with hierarchical branched structures that mimic native vascular architecture. The processed templates were then embedded within different matrices (PDMS/PCL/PEGDA/agarose/silk fibroin/fibrin) and subsequently removed by dissolution in aqueous media. The channel resolution achieved in this study was ≈ 300 µm, with the structural fidelity well‐preserved following endothelial cell seeding (Figure 7K). The vascular networks were shown to support cell viability and metabolic activity in thick, tissue‐like constructs through perfusion. Furthermore, the networks enabled the perfusion of hepatic constructs and supported the engraftment of vascularized tissue in animal models.

The SLS technology excels at fabricating suspended and seamless branched structures, as its layer‐by‐layer fusion process enables the creation of intricate, continuous networks without joints. However, its resolution tends to be inferior to other technologies, due to limitations in the size of the powder particles and the precision of the laser.

#### 5.3.5 MEW

MEW prints sacrificial materials by extruding a molten polymer through a fine nozzle while applying a high‐voltage electric field to precisely guide fiber deposition.^[^ ^195^ ^]^ PCL was used as a sacrificial material and printed via MEW to create vascular structures embedded in HA methacrylate (HAMA) hydrogels.^[^ ^86^ ^]^ Following UV crosslinking, PCL was selectively removed through a stepwise solvent exchange process with acetone and dichloromethane, resulting in perfusable microchannels. This approach successfully produced 10 µm vascular channels after shrinking, showing exceptional resolution enhancement. Additionally, a 300 µm endothelialized vascular construct was demonstrated, further highlighting the potential of this approach for fabricating microvasculature.

A print‐and‐fuse strategy was introduced to generate perfusable microvascular networks within GelMA hydrogels.^[^ ^64^ ^]^ Sacrificial templates were fabricated via MEW using thermoresponsive PcycloPrOx. Given its LCST of 25 °C, the polymer swelled when embedded in GelMA solution at temperatures above this threshold, inducing the fusion of adjacent filaments and formation of a continuous branched architecture (Figure 7L). After UV crosslinking of GelMA hydrogel, the entire structure was placed at a temperature below 25 °C, enabling complete dissolution of the sacrificial material and yielding interconnected microchannels. With an initial fiber diameter of 87–275 µm, the swelling effect (1.3–1.5× expansion) resulted in final microchannel dimensions of ≈110–410 µm. The vascular network followed Murray's law, exhibiting biomimetic branching characteristics, with the endothelialized lumen reaching 400 µm. Functional validation, including barrier integrity assays, permeability studies and tumor necrosis factor alpha stimulation, demonstrated the system's potential for vascularized tissue engineering and organ‐on‐a‐chip applications.

The MEW technology integrates DIW and electrospinning. Compared to DIW, the introduction of an electric field significantly improves resolution, enabling the production of ultrafine fibers. In contrast to electrospinning, MEW allows for controlled fiber alignment, ensuring greater structural control. Yet, the need for high temperatures to maintain polymer flow and high voltages to control deposition may adversely affect biocompatibility and cell viability, limiting its suitability for certain biomedical applications.

### 5.4 Bioprinting

Bioprinting is an advanced form of 3D printing technology that utilizes bioinks composed of living cells and/or biomaterials to fabricate tissue‐like structures.^[^ ^196^ ^]^ Unlike traditional 3D printing, which typically processes inert materials such as polymers or metals, bioprinting is specifically designed to create biologically functional constructs, making it particularly suited for applications in tissue engineering and regenerative medicine. It is worth noting that the 3D printing approaches described in the previous section can also be potentially used for bioprinting when the inks are mixed with cells during the printing processes, with further cytocompatibility optimizations. Among these technologies, co‐axial printing is frequently employed in bioprinting to fabricate multilayered vascular structures with different cell types distributed in each layer, aiming to mimic the native organizations and cellular heterogeneity of blood vessels. In sacrificial biofabrication, bioprinting offers a unique advantage by directly printing sacrificial materials pre‐encapsulated with endothelial cells into defined geometries (Figure 7M). This approach eliminates the need for subsequent seeding of endothelial cells, thereby reducing the risk of blockages or uneven cell distributions within the bioprinted channels, ensuring better structural and functional uniformity.

Gelatin was used as a sacrificial material to create vascular structures within thick scaffolds by the bioprinting technology.^[^ ^49^ ^]^ Gelatin mixed with HUVECs and collagen matrix was bioprinted to form predefined fluidic channels. The bioprinting process was conducted under precise and controlled conditions to ensure structural fidelity and cell viability. After bioprinting, collagen matrix was gelled using nebulized sodium bicarbonate, and the constructs were warmed to 37 °C to remove sacrificial gelatin, finally forming vascular networks with endothelial lining. The diameter of the microchannels was ≈ 500 µm. The seeded HUVECs adhered, migrated, and spread along the channel walls, which produced functional, endothelialized lumens capable of sustaining dynamic perfusion. Later, Stevens and co‐workers proposed a void‐free 3D bioprinting (VF‐3DP) technology by printing sacrificial bioink and matrix bioink together.^[^ ^177^ ^]^ Gelatin, also used as a sacrificial material in this study, was combined with endothelial cells and co‐printed in a layer‐by‐layer manner with matrix bioink containing GelMA and tissue cells. Following UV crosslinking of GelMA and removal of gelatin at 37 °C, well‐defined endothelialized vascular channels with a diameter of 450 µm were developed. The constructs were demonstrated to effectively support endothelial cell viability, proliferation, and barrier functions.

The co‐axial method was combined with bioprinting to fabricate vascularized tubular constructs using gelatin as sacrificial material.^[^ ^178^ ^]^ The inner core of the bioprinted structure consisted of 5% gelatin loaded with HUVECs, while the outer sheath was composed of gelatin‐PEG‐tyramine (GPT) bioink loaded with human dermal fibroblasts. The sacrificial gelatin core was rapidly dissolved at 37 °C within 5 min, leaving behind hollow channels with diameters of ≈485 µm. Following 8 days of in vitro culture, the channels were successfully endothelialized. In vitro assessments confirmed high cell viability (>90%) and cellular elongation. He and co‐workers replaced the shell layer with GelMA and fabricated 200 µm endothelialized channels by precisely adjusting needle sizes and flow rates.^[^ ^179^ ^]^ This approach enabled the successful fabrication of 3D cell‐laden vascularized tissue constructs (≥1 cm) that sustained long‐term cell culture (≥20 days). This method also generated vascularized cancer and osteogenic tissue constructs, demonstrating great potential in angiogenesis research, nutrient transport, and regenerative medicine.

In previous research on bioprinting technology, gelatin was used as a sacrificial material owing to its ability to be removed in situ under physiological conditions without causing harm to encapsulated cells. However, the low printing resolution of gelatin typically produces cell‐laden constructs with diameters of ≈500 µm, posing significant challenges for fabricating microvascular networks below 100 µm. Furthermore, the high shear stress encountered during extrusion may damage encapsulated cells, further restricting its applicability. In 2024, we pioneered the use of DNA hydrogel as a sacrificial material in the bioprinting technology, which was termed the printing cell embedded sacrificial strategy (PRINCESS), and successfully generated endothelialized microvascular networks with a diameter as small as 70 µm, breaking the current restrictions of 100 µm.^[^ ^41^ ^]^ This is the smallest endothelialized microvascular structure fabricated by bioprinting to date. DNA hydrogel offers several unique advantages.^[^ ^197^ ^]^ It is enzymatically degradable under physiological conditions, providing rapid and controllable biodegradability. Its exceptional shear‐thinning property makes it suitable as a biolubricant, protecting cells from mechanical damage during the bioprinting process.^[^ ^142^ ^]^ Additionally, the self‐healing property of DNA hydrogel enables the fabrication of intricate and branched structures.^[^ ^198^ ^]^ Therefore, DNA hydrogel is a favorable sacrificial material for the PRINCESS approach. Using a custom‐designed 3D bioprinter equipped with capillary‐based nozzles, DNA hydrogel was printed into fine filaments with a resolution of 25 µm. Finally, DNA biolubricant was embedded with HUVECs and bioprinted to form a 70‐µm endothelialized microvasculature, which exhibited robust endothelial cell adhesion along the channel walls, forming well‐defined lumen structures. Moreover, by encapsulating hepatocytes in the surrounding material, vascularized liver tissue models were also fabricated (Figure 7M), highlighting the potential of this method for advancing organ‐on‐a‐chip systems and tissue engineering.

Thomas and co‐workers combined DLP printing with the bioprinting technology to fabricate vascularized constructs.^[^ ^104^ ^]^ The enzymatically degradable sacrificial bioink, HAMA, was mixed with HUVECs and supporting cells (e.g., 10T1/2 cells), and then photopolymerized during bioprinting to create designed patterns. Following casting GelMA surrounding scaffolds, hyaluronidase was applied to selectively degrade the HAMA bioink, releasing the encapsulated cells and forming hollow, perfusable vascular channels. The smallest diameter of fabricated vascular channels was 360 µm. The encapsulated endothelial cells adhered to channel walls, proliferated, and self‐assembled into confluent endothelial linings, which were able to sustain over 28 days.

The bioprinting technology obviates the need for perfusion‐based endothelialization, achieving density and spatial regulations of seeded cells and leading to high reproducibility. However, bioprinting cannot eliminate the need for perfusion culture entirely, which is still required when the attached endothelial cells need to continuously grow and proliferate.^[^ ^41^ ^]^ Meanwhile, this method imposes stringent requirements on sacrificial materials, which need to have cytocompatibility for effective cell encapsulation, shear‐thinning property for cell protection during the 3D bioprinting process, self‐healing property for the formation of branched structures, and controllable decrosslinking property for the achievement of in‐situ sacrifice after printing.

### 5.5 Discussions of Technologies

The aforementioned fabrication technologies provide powerful tools for sacrificial biofabrication across different biomedical applications. Since native blood vessels span a wide diameter spectrum from ≈1 µm–25 mm,^[^ ^2^ ^]^ the range of fabrication dimensions is a critical factor in vascular construction. An ideal fabrication technology must combine high spatial resolution with the capacity to construct macroscale architectures. Here, the fabrication diameters of endothelialized vasculature for each technology are summarized in Figure 7. The spinning technology exhibits superior resolution, enabling the formation of endothelialized vasculature as small as 10 µm.^[^ ^93^ ^]^ However, its utility is limited to microscale constructs and is unsuitable for fabricating larger vessels. In contrast, molding, 3D printing, and bioprinting technologies support broader scalability and can generate large‐diameter structures, yet their resolutions typically fall in the range of 30–70 µm.^[^ ^41^, ^56^, ^91^ ^]^ Notably, the resolution of bioprinting is currently constrained to 70 µm due to the damaging effects of shear stress on encapsulated cells during high‐resolution printing.^[^ ^41^ ^]^ Given the hierarchical and multiscale nature of the human vasculature, a single technology is insufficient to recapitulate the dimensional complexity. Therefore, the integration of existing fabrication technologies is essential. Such hybrid strategies can compensate for the limitations of individual technologies and enable the fabrication of vascular constructs that mimic the structural gradients observed in native tissues.

In addition, native arteries and veins usually possess well‐defined multilayered structures,^[^ ^5^ ^]^ yet most current fabrication technologies are restricted to producing capillary‐like constructs comprising only a single layer of endothelial cells. The approaches that have been taken to engineer vascular structures with different layers are less frequently reported. One approach is co‐axial bioprinting, which enables the deposition of concentric tubular structures by encapsulating different cell types in distinct regions.^[^ ^48^, ^174^ ^]^ The other approach involves sequential immersion and crosslinking steps to build multiple layers around the sacrificial templates.^[^ ^171^ ^]^ While both techniques offer structural and compositional resemblance to native vessels, the specific biological functions associated with each layer remain to be demonstrated. Further research is therefore needed to achieve functionally biomimetic multilayered vasculature.

In summary, sacrificial biofabrication technologies currently achieve channel resolutions down to 10 µm and support the preparation of multilayered structures incorporating different cell types. Despite these advances, clinical translation will require submicron precision for replicating capillary networks and functionally biomimetic multilayered vasculature, which remains a challenge.

## 6 Applications

**Figure 8:**
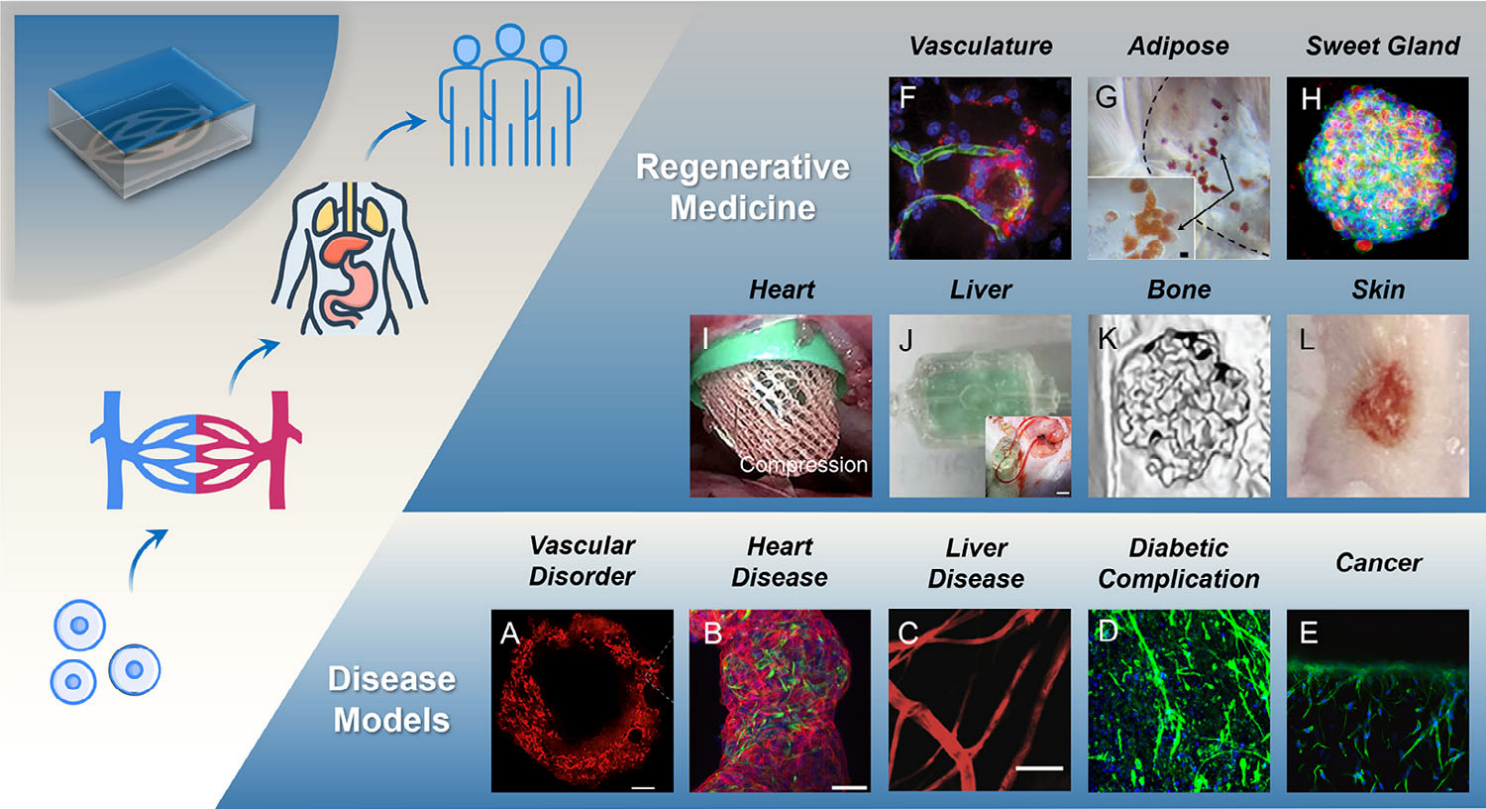
Applications of sacrificial biofabrication in disease models and regenerative medicine. The left part illustrates that sacrificial biofabrication creates biomimetic scaffolds with endothelialized vasculature, which can be used to prepare functional organs and tissues, ultimately improving human health. Disease models include A) Vascular disorder: fluorescently labeled 3D‐printed model of the pulmonary arterial adventitia. Reproduced with permission.^[^ ^50^ ^]^ Copyright 2022, IOP Publishing. B) Heart disease: immunofluorescence staining for cardiac troponin‐T and vimentin in healthy cardiac microtissue within the surrounding hydrogel. Reproduced with permission.^[^ ^101^ ^]^ Copyright 2021, Springer Nature. C) Liver disease: fluorescence image of perfused microchannel network to mimic the complex capillary network of the liver. Reproduced with permission.^[^ ^62^ ^]^ Copyright 2019, Wiley‐VCH. D) Diabetic complication: confocal microscope image of the in vitro type 2 diabetic human skin model. Reproduced with permission.^[^ ^201^ ^]^ Copyright 2021, IOP Publishing. E) Cancer: F‐actin and CD31 staining of the lymphatic endothelial cells in the evaluation model for lymphangiogenesis. Reproduced with permission.^[^ ^202^ ^]^ Copyright 2021, Elsevier. Regenerative medicine includes F) Vasculature: fluorescence image of vascular patches in hind limb ischemia. Reproduced with permission.^[^ ^77^ ^]^ Copyright 2017, Springer Nature. G) Adipose: Oil Red O staining of human mesenchymal stem cells seeded in adipogenic differentiation medium. Reproduced with permission.^[^ ^98^ ^]^ Copyright 2019, Elsevier. H) Sweet gland: identification of the cell viability and lineage of induced sweat gland cell spheroids. Reproduced with permission.^[^ ^87^ ^]^ Copyright 2023, Elsevier. I) Heart: delivery of the printed epicardial device through the trocar. Reproduced with permission.^[^ ^74^ ^]^ Copyright 2021, Springer Nature. J) Liver: images of channeled tissue construct before and after establishing anastomosis with the artery and vein. Reproduced with permission.^[^ ^51^ ^]^ Copyright 2021, Wiley‐VCH. K) Bone: 3D reconstructed micro‐CT image in the defect site of transverse section after printed scaffold implantation for 12 weeks. Reproduced with permission.^[^ ^75^ ^]^ Copyright 2022, Wiley‐VCH). L) Skin: photograph of wound healing site after implantation of the microchannel network hydrogel. Reproduced with permission.^[^ ^63^ ^]^ Copyright 2020, Springer Nature.

Sacrificial biofabrication has been proven to be an effective approach for engineering vasculatures within tissue constructs, which has been widely used in different fields of biomedicine, such as disease modeling and regenerative medicine (**Figure** **8**).

### 6.1 Disease Models

Disease progression has been shown to be intricately connected to the vascular networks within tissues. In disease models, the sacrificial biofabrication enables the replication of realistic vascular systems, providing more accurate environments for studying disease mechanisms and toxicity screening.^[^ ^199^ ^]^ This approach facilitates the testing of therapeutic interventions under conditions that closely mimic the human body. As a result, it offers more reliable predictions of treatment efficacy while also reducing the reliance on animal models.^[^ ^200^ ^]^ By recapitulating tissue complexity, in vitro models enable physiologically relevant modeling of human developmental processes and disease mechanisms, while offering scalable platforms for assessing drug toxicity and therapeutic efficacy.

#### 6.1.1 Vascular Disorders

Advanced engineered platforms have been established to recapitulate pathological mechanisms of vascular disorders, including stenosis/atherosclerosis, intimal hyperplasia, pulmonary hypertension, and thrombosis. For example, therapeutic interventions for pulmonary hypertension are limited in part due to the lack of in vitro screening platforms that accurately reproduce dynamic arterial wall mechanical properties.^[^ ^203^ ^]^ A 3D‐printed, light‐adjustable model of the outer membrane of the pulmonary artery was designed to study the pathogenesis of fibrosis in vitro using the embedded printing technology (Figure 8A).^[^ ^50^ ^]^ The hydrogel platform showed an initial elastic modulus in the range of healthy pulmonary artery tissue, hardening into the pathological range of hypertensive tissue, and supporting cell proliferation over time. The cell culture platform could accurately mimic the native and pathologic pulmonary arterial adventitial microenvironment.

#### 6.1.2 Heart Diseases

Progress in vascularization technologies now allows the creation of in vitro models and implants that more accurately replicate the intricate structure of natural tissues and organs. The integration of vascularized constructs in cardiac disease modeling is critical for replicating nutrient exchange and cellular crosstalk within ischemic myocardium. Spheroids fabricated by embedded printing within dual‐component hydrogels were presented for constructing high‐cell‐density heterogeneous tissue models (Figure 8B).^[^ ^101^ ^]^ Using shear‐thinning HA hydrogel as a support matrix, the method achieves high‐precision positioning of multicellular spheroids with 95% viability. By adjusting induced pluripotent stem cell‐derived cardiomyocyte/fibroblast ratios, the printed models replicate pathological features of myocardial infarction scars, including reduced contractility and impaired electrical signal synchronization. The platform demonstrated therapeutic evaluation capabilities, showing micro ribonucleic acid (miRNA) treatment enhanced cardiomyocyte proliferation and improved scar synchronization, tripling contraction amplitude.

#### 6.1.3 Liver Diseases

Liver models are increasingly vital for drug metabolism studies and foodborne illness screening, as endorsed by the U.S. Food and Drug Administration (FDA). However, challenges persist in engineering perfusable vascular networks (≤200 µm spacing) and replicating progressive liver diseases. We previously engineered a vascularized liver construct incorporating perfusable endothelialized channels using agarose as the sacrificial material, along with the embedded printing technology.^[^ ^204^ ^]^ This endothelialized model addressed critical gaps in conventional hepatotoxicity screening by recapitulating the vascular‐tissue crosstalk, enabling precise evaluation of first‐pass metabolism and chronic exposure effects. A 3D perfusable liver model for disease modeling and drug testing was also fabricated (Figure 8C).^[^ ^62^ ^]^ The system utilized sacrificial PNIPAM fibers fabricated by the solvent spinning technology embedded within gelatin hydrogels to recreate a vascular network, enabling effective perfusion and dense hepatocyte spheroid culture. This model successfully recapitulated the progression of nonalcoholic fatty liver disease, including steatosis, inflammation, and fibrosis. Therapeutic effects, such as mitochondrial recovery and reduced oxidative stress following Ezetimibe treatment, were accurately predicted. With its scalability for high‐throughput drug screening and potential applications in organ therapy, this platform offered a reliable alternative to animal models for liver disease research.

#### 6.1.4 Diabetic Complications

Diabetes, a metabolic disorder characterized by chronic high blood sugar, manifests in subtypes with distinct underlying causes. It substantially increases risks for severe complications such as kidney failure, cardiovascular disease, stroke, vision loss, and foot ulcers. To study these effects, 3D models have emerged as powerful tools for drug testing. The ECM property of the retina plexiform layer was studied based on a 3D‐printed coculture model.^[^ ^205^ ^]^ The results showed that high glucose levels influenced both endothelial cells and microglia phenotypes, gene expressions, and angiogenic potential, which highlighted the importance of cell interactions and microenvironmental factors in diabetic retinopathy therapy and aimed to identify new strategies and improve understanding of the role of microglia in disease pathogenesis. Kenar and co‐workers successfully developed a novel 3D diabetic human skin model that integrates primary cells from diabetic patients with biomaterial technology to precisely simulate diabetic skin pathology (Figure 8D).^[^ ^201^ ^]^ The model features stratified implantation of patient‐derived dermal fibroblasts, vascular endothelial cells, and keratinocytes, forming a 1.86 mm‐thick skin structure under hyperglycemic conditions. Functional validation demonstrated its vascularization capability, forming dermal papilla‐like capillary networks, and tracked fibroblast migration in wound healing assays. It provides a physiologically relevant platform for screening antidiabetic drugs and evaluating wound‐healing materials.

#### 6.1.5 Cancers

In vitro cancer tissue models provide an efficient platform for precision oncology research by preserving tumor biology and mimicking the microenvironment in vivo.^[^ ^206^ ^]^ A 3D‐printed breast tumor model with integrated lymphatic vessels was developed to study cancer‐lymphatic interactions.^[^ ^202^ ^]^ Using sacrificial agarose microfibers printed by the DIW technology, hollow channels seeded with lymphatic endothelial cells within GelMA hydrogels containing breast cancer cells were created (Figure 8E). Cancer cells migrated toward lymphatics, adopting invasive shapes near vessels. The non‐toxic sacrificial method allowed precise lymphatic architecture control. It enabled mechanistic studies of metastasis, anti‐lymphangiogenic drug testing, and adaptation for other cancers. Satchi‐Fainaro and co‐workers proposed glioblastoma models to replicate the intricate tumor microenvironment.^[^ ^57^ ^]^ The model integrated glioblastoma cells, astrocytes, and microglia into fibrin bioinks, with perfusable vascular networks created by the DIW technology using Pluronic F‐127 as the sacrificial material. This model accurately mimics tumor growth, invasion, and therapeutic resistance, showing similar genetic signatures and drug responses as in vivo mouse models. The ability to evaluate tumor‐stroma interactions and drug efficacy highlights its potential for advancing preclinical drug screening and personalized medicine. Another study introduced vascularized tumor‐on‐a‐chip models for neuroblastoma research based on DIW printed Pluronic F‐127 as the sacrificial template.^[^ ^58^ ^]^ Neuroblastoma spheroids were embedded within fibrin‐GelMA matrix alongside perfusable vascular networks formed by endothelial cells and mesenchymal stem cells. Key tumor behaviors such as angiogenesis, tumor spheroid growth, and early metastatic cell migration were replicated using this platform. This model enabled detailed exploration of tumor‐microenvironment interactions, holding significant promise for personalized cancer research and preclinical drug screening, particularly for high‐stage neuroblastoma.

On the other hand, circulating tumor cell (CTC) behavior during metastasis was studied using hydrogel‐based vascular flow devices.^[^ ^59^ ^]^ The 3D‐printed devices incorporating endothelialized channels were constructed by the DIW technology with sacrificial Pluronic F‐127 templates, which replicate vascular geometries and enable precise control of CTC dynamics under physiological flow conditions. Using fluorescently labeled tumor cells, the in vitro model revealed preferential CTC adhesion at vascular branch points and highlighted differences between acellular and endothelialized environments. Additionally, the platform validated the accuracy of computational flow models, demonstrating its utility for investigating biophysical forces during metastasis. The device offers a versatile tool for exploring cancer biology and evaluating therapeutic interventions in a controlled vascular environment. Cancer extravasation in the brain was also studied with a 3D‐printed multilayered cerebrovascular conduit.^[^ ^60^ ^]^ This study combined embedded printing and coaxial printing to fabricate the brain metastasis models. The core‐shell tubular structures were printed within a cell‐laden bath, with the Pluronic F‐127 core serving as the sacrificial material and the intermediate and outer layers loaded with endothelial and perivascular cells, respectively. This system effectively replicated tumor cell adhesion, extravasation, and invasion in vitro. Tumor cells showed preferential adhesion at curved vascular regions, mimicking metastatic patterns observed in vivo. This platform provided a novel approach to study the biomechanical and molecular mechanisms in brain metastasis, with potential applications in cancer research and drug testing.

### 6.2 Regenerative Medicine

Sacrificial biofabrication is pivotal in regenerative medicine for engineering pre‐vascularized networks within biomimetic scaffolds, addressing a critical barrier in organ regeneration: nutrient and oxygen deprivation.^[^ ^207^ ^]^ Large organs, with their dense cellular architectures, often fail due to rapid necrosis caused by insufficient vascularization. By embedding perfusable vascular channels during scaffold fabrication, these strategies ensure sustained delivery of oxygen, nutrients, and growth factors to deep tissue layers, mimicking native vascular physiology. When applied to wounds or damaged organs, the engineered vasculature not only prevents ischemic necrosis but also recruits endogenous cells to enhance repair quality, reducing fibrosis and scarring. Beyond localized repair, such vascularized scaffolds enable integration with host circulatory systems‐a prerequisite for functional organ transplantation. This dual functionality, preventing necrosis during healing and enabling host integration, positions the sacrificial biofabrication as a cornerstone for scalable, clinically viable organ regeneration.^[^ ^208^ ^]^

#### 6.2.1 Vasculature

Chen and co‐workers designed 3D‐printed vascular patches with endothelial cell‐lined channels to promote therapeutic angiogenesis for tissue repair in ischemic environments (Figure 8F).^[^ ^77^ ^]^ The patches integrated patterned microvascular networks fabricated by the FDM technology using carbohydrate glass as the sacrificial material in fibrin hydrogel scaffolds, enhancing vascular connectivity and perfusion. Endothelial cells within the constructs exhibited robust alignment and network formation. In vivo, using rodent hind limb ischemia models, the patches significantly restored blood flow, mitigated muscle atrophy, and preserved tissue function through direct anastomosis with host vasculature. Laser Doppler imaging and histological analysis confirmed increased capillary density and functional perfusion. This approach provides a scalable platform for enhancing tissue repair and recovery in ischemic diseases.

#### 6.2.2 Adipose Tissue

Marelli and co‐workers presented a gelatin‐based scaffold employing sacrificial alginate templates for adipose tissue regeneration (Figure 8G).^[^ ^98^ ^]^ The scaffold integrates microporosity and prevascularization through the incorporation of alginate microbeads and DIW printed filaments, respectively. Human mesenchymal stem cells embedded in the scaffold showed cell proliferation, adipogenic differentiation, and lipid droplet formation in vitro. The scaffold was also confirmed to support blood flow through engineered channels following anastomosis with rat vasculature. By mimicking the mechanical and structural properties of native adipose tissue, the scaffold facilitates cellular infiltration and vascular integration, making it a promising candidate for organ and tissue regeneration applications.

#### 6.2.3 Sweat Gland

Fu and co‐workers reported biomimetic models for sweat gland regeneration by combining advanced 3D printing technology with a prevascularized ECM.^[^ ^87^ ^]^ PCL was used as the sacrificial material to generate vascular structures by the melt spinning technology. Adipose‐derived mesenchymal stem cells and dermal microvascular endothelial cells were used to generate sweat gland‐like spheroids, which were subsequently seeded into the vascularized ECM (Figure 8H). In a thermal‐injury mouse model, the transplantation of these vascularized spheroids successfully restored sweat gland functions, as demonstrated by increased sweat pore activity in functional assays. The constructs promoted reciprocal interaction between glandular and vascular cells, facilitating glandular morphogenesis and vascular integration. This innovative approach offers great potential for functional sweat gland regeneration and broad applications in skin repair.

#### 6.2.4 Heart

Ye and co‐workers developed a multifunctional epicardial device (PerMed) for heart repair following myocardial infarction (Figure 8I).^[^ ^74^ ^]^ The device integrates a biodegradable elastic patch to provide mechanical support to the infarcted myocardium and permeable hierarchical microchannel networks fabricated by the FDM technology using sacrificial sugar templates to promote angiogenesis and cell infiltration. The patch demonstrated enhanced cardiomyocyte viability and adhesion, while in vivo experiments in both rat and porcine models showed improved left ventricular function, reduced fibrosis, and enhanced vascularization. Additionally, a connected pump allowed for sustained delivery of therapeutic agents, further amplifying repair effects.

Biomimetic scaffolds designed for cardiac regeneration were also developed by incorporating oriented micropores and a branched vascular channel network.^[^ ^78^ ^]^ This study utilized carbohydrate glass as the sacrificial material that was fabricated by the FDM technology to generate the vascular network. With cardiomyocytes and endothelial cells seeded in distinct zones, the scaffold supported synchronized beating of engineered cardiac tissue under electrical stimulation, indicating functional integration. The scaffold promoted cell alignment, sarcomere formation, and transport of nutrients and oxygen. This architecture replicated the anisotropic structure and vascularization of native myocardium, addressing challenges in creating vascularized cardiac tissue.

#### 6.2.5 Liver

Gu and co‐workers study proposed a printed liver tissue model tailored for applications in liver regeneration (Figure 8J).^[^ ^51^ ^]^ The model incorporated branched vascular networks fabricated through DIW printing using sacrificial gelatin templates, enabling efficient mass transport. In vitro experiments demonstrated functional hepatocyte activities, including the synthesis of liver‐specific proteins, while co‐culture systems with endothelial cells facilitated vascularization. In vivo, the constructs maintained structural integrity and vascular connectivity upon surgical anastomosis with host vasculature, promoting tissue integration and neovascularization. Later, they presented a novel microgel‐hydrogel hybrid (GMMHM) as a mechanically robust and biologically supportive scaffold for 3D printing of vascularized, high‐cell‐density liver tissue.^[^ ^52^ ^]^ A gelatin‐based sacrificial ink was employed to create perfusable macrovascular channels by embedded printing, and the GMMHM surrounding material, composed of GelMA microgels, HAMA, and Matrigel, enables structural stability under physiological shear stress while promoting endothelialization and vascular morphogenesis. This engineered construct was successfully implanted into rat models with 85% hepatectomy, achieving direct vascular anastomosis, rapid hepatic function compensation, and improved survival, which demonstrated its translational potential for acute liver failure therapy.

#### 6.2.6 Bone

Bone regeneration^[^ ^209^ ^]^ was achieved using nanofibrous scaffolds with hierarchical microstructures and dual‐drug delivery systems (Figure 8K).^[^ ^75^ ^]^ By employing FDM printed sacrificial sugar materials, the scaffold integrated interconnected microchannels to enhance nutrient diffusion, cell migration, and vascular integration. In critical‐sized skull defect models in rats, the scaffold demonstrated sequential release of dimethyloxalylglycine, a pro‐angiogenic agent, and bone‐forming peptide‐1, an osteogenic factor, to synergistically promote angiogenesis and osteogenesis. Cell experiments validated the enhanced endothelial cell migration, tube formation, and osteoblast differentiation, confirming its capacity to support vascularization and mineralization. Rapid neovascularization and significant new bone formation were observed in vivo, with defect areas nearly entirely covered by regenerated bone after 12 weeks, which provides a robust platform for addressing large‐scale bone defects.

#### 6.2.7 Skin

Wound healing has been demonstrated using microchannel‐network hydrogels, which featured 3D interconnected channel structures generated by solution spinning of the sacrificial PNIPAM material to promote vascularization and tissue regeneration (Figure 8L).^[^ ^63^ ^]^ Endothelial cell infiltration, cell migration, angiogenic activity, and tube formation were enhanced in vitro. The hydrogels were applied to full‐thickness dorsal skin wounds in mice, where it accelerated wound closure, increased collagen deposition, and promoted epidermal regeneration. Histological analysis revealed extensive vascular ingrowth and improved tissue architecture. This approach highlights a structurally guided mechanism for enhancing wound repair, providing a scalable and effective strategy for tissue regeneration.

To date, sacrificial biofabrication has been widely applied in disease modeling and regenerative medicine, and its applications may further extend to implantable sensors and drug delivery. For clinical application, major challenges remain in achieving surgical integration with host vasculature through anastomosis while simultaneously minimizing immune response.

## 7 Perspectives

**Figure 9:**
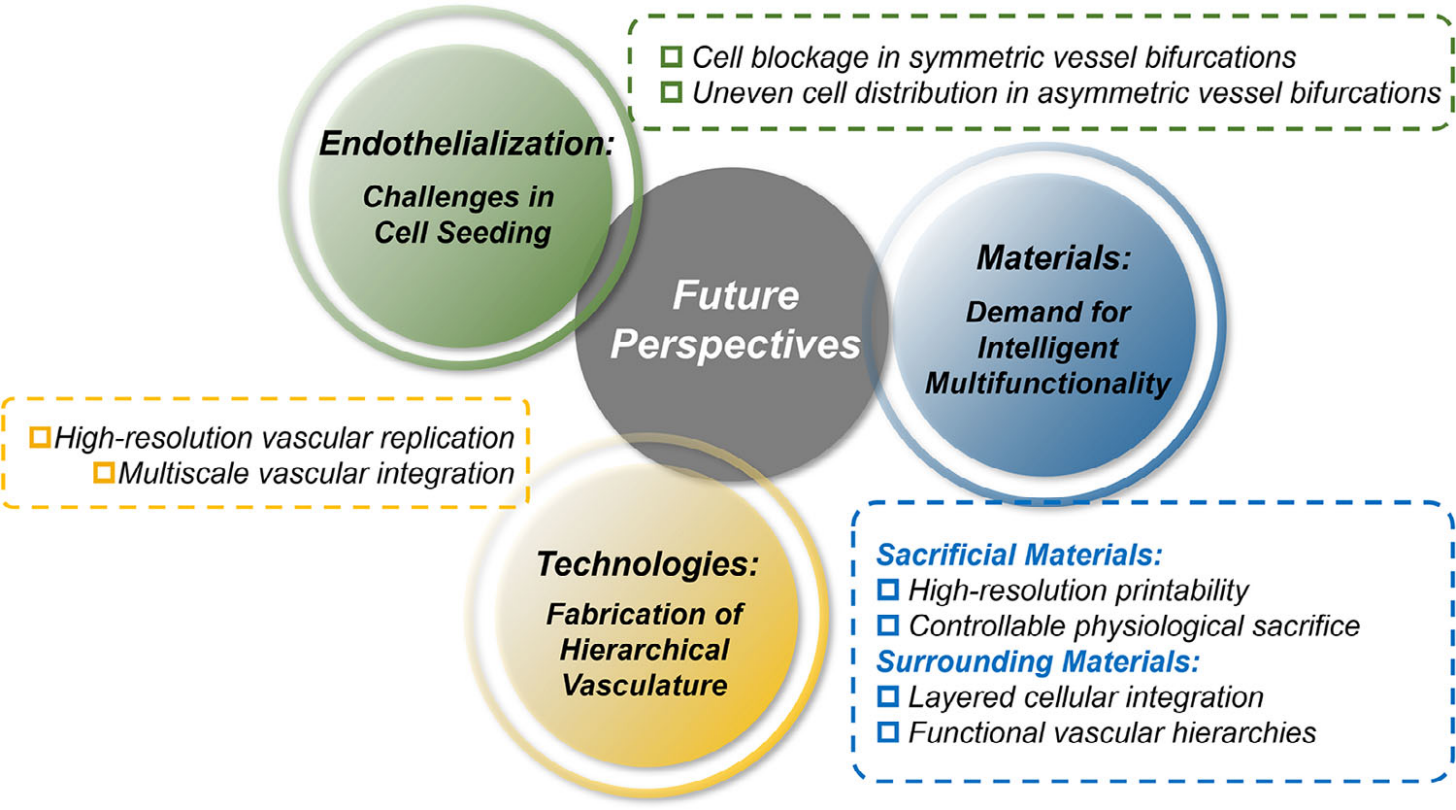
Future perspectives of the sacrificial biofabrication for vascularization in aspects of endothelialization, materials, and technologies.

The sacrificial biofabrication serves as an effective tool in biomedical engineering, providing a viable solution for building functional vasculatures. This strategy facilitates the development of intricate, perfusable vascular systems, addresses many of the limitations associated with traditional strategies. Although numerous sacrificial materials, surrounding materials, fabrication technologies, and biomedical applications have been reported, several critical and challenging areas remain to be thoroughly investigated (**Figure** **9**).

### 7.1 Endothelialization: Challenges in Cell Seeding

Endothelialization is essential for the formation, integrity, and functionality of the vasculature. Endothelial cells exhibit diverse phenotypes that correspond to the different structures and metabolic requirements of each tissue and organ.^[^ ^210^ ^]^ Due to this heterogeneity, utilizing organ‐specific endothelial cells in vitro is essential for understanding their roles in disease progression and enhancing the relevance of drug testing results from laboratory studies to real‐world situations. To develop meaningful human‐based in vitro models that are valuable for drug development, testing, or analyzing early disease stages, it is crucial to understand the heterogeneity of endothelial cells and choose the corresponding types.

In addition to the bioprinting technology, all the other technologies introduced above, including the decellularized organ strategy, are based on forming the channels and subsequently seeding them with endothelial cells. Cell seeding technologies include static seeding and dynamic seeding.^[^ ^40^ ^]^ Static seeding, meaning pipetting a cell suspension into the scaffold directly, is the simplest and most widely used method, but it suffers from low seeding efficiencies. To enhance efficiency and uniformity during static seeding, biological glue such as fibrin has been employed to promote the adhesion of cells on the scaffold. Compared to static seeding, dynamic seeding offers improved seeding efficiency and uniformity. Common dynamic seeding methods include rotational seeding^[^ ^211^ ^]^ and vacuum seeding.^[^ ^212^ ^]^ By integrating some of these dynamic seeding methods, hybrid systems such as rotational vacuum seeding and perfusion bioreactor systems have been developed to further increase cell seeding efficiency.^[^ ^213^, ^214^ ^]^ Other methods include sheet‐based cell seeding, which can work without the use of foreign materials^[^ ^215^ ^]^; electrostatic and magnetic cell seeding, which exploit physical forces to help cells distribute^[^ ^216^, ^217^ ^]^; and photopolymerized hydrogels, which show potential in spatial cell encapsulation.^[^ ^218^ ^]^ Despite these advances, there remain challenges in cell seeding for vascularization. For example, the fate of the cells post‐seeding is usually not clear, and how to scale up current methods for clinical use remains a concern.

**Figure 10:**
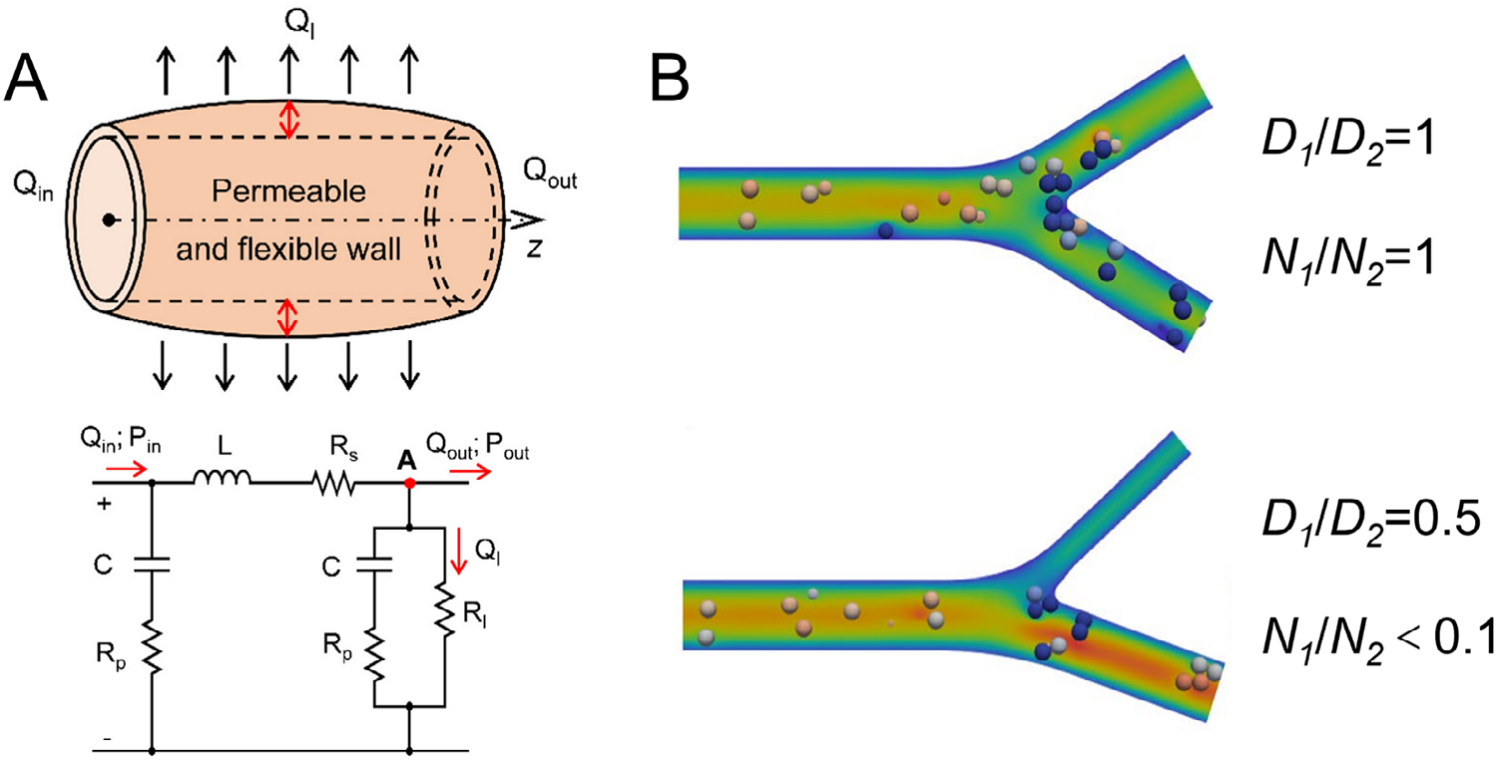
Simulation of the cell seeding process in engineered blood vessels. A) Schematic illustration of permeable flow in a blood vessel and its equivalent π‐filter circuit. Reproduced with permission.^[^ ^219^ ^]^ Copyright 2023, Elsevier. B) Stimulation of cell distributions in symmetrical and asymmetrical vessel bifurcations. Reproduced with permission.^[^ ^220^ ^]^ Copyright 2023, Springer Nature.

The fluid behaviors of the cell seeding process significantly influence the uniformity of endothelialization. To further investigate this process, Nguyen and co‐workers employed the electrical analog permeability (EPA) model to simulate the permeable flow characteristics in the vessels.^[^ ^219^ ^]^ As illustrated in **Figure** **10**A, based on the analogy between fluid flow and electricity flow, the fluid flow in vascular networks is modeled as a multi‐stage π‐filter circuit, enabling the analysis of pressure distribution and flow characteristics. Key vascular properties are analogous to electric components: the hydraulic resistance of fluid flow within the vessel lumen is represented by *R_s_* (resistor), which is related to the length, diameter, and fluid viscosity of blood vessels. The inertial effects of the fluid are defined as *L* (inductor). The elasticity of the vessel during the pressure changes is captured by *C* (capacitor). Furthermore, the viscoelastic characteristics of the vessel wall are described by *R_p_* (parallel resistor), while the permeability of the vessel walls is reflected by *R_l_​* (leakage resistor). Therefore, the fundamental equations of fluid flow through a blood vessel can be expressed as follows:

(1) $$C\frac{dP}{dt}+Q_{\mathrm{out}}-Q_{\mathrm{in}}+CR_{p}\left(\frac{dQ_{in}}{dt}-\frac{dQ_{out}}{dt}\right)+\frac{1}{R_{1}}P=0$$

(2) $$L\frac{dQ}{dt}+P_{\mathrm{out}}-P_{\mathrm{in}}+R_{s}Q=0$$

Here, 𝑃 represents the fluid pressure, 𝑄 denotes the fluid flow rate, and 𝑡 refers to time. The subscripts “in” and “out” respectively correspond to the variables at the inlet and outlet.

Combined with the coupled computational fluid dynamics (CFD)‐discrete element method (DEM) model, the EPA model was used to stimulate the flow behavior and clogging mechanisms of cell suspensions.^[^ ^220^ ^]^ The study reveals that when the diameter of the vessel is smaller than the cell diameter (typically 18 µm), the cell suspension fails to flow effectively into the channel, primarily due to the sieving effect that leads to cell aggregation and clogging. Moreover, in the presence of branched vessels, the distribution of cells varies based on branch symmetry (Figure 10B). In symmetric vessel bifurcations, cells tend to distribute evenly. However, cells are more likely to adhere at the bifurcation apex, resulting in successive accumulation and arch formation. In contrast, in asymmetric vessel bifurcations, fluid and cell flow are governed by the Zweifach‐Fung effect, where the majority of cells preferentially flow into the branch with the larger diameter. Notably, when the diameter ratio is 0.5, the smaller branch receives almost no cell inflow (less than 10%), leading to highly uneven cell distribution. Consequently, strategies for subsequent cell seeding into channels are generally applicable only to symmetric structures, and the channel diameter has to be sufficiently large to prevent cell clogging.

The flows within blood vessel lumens always generate shear stresses, which have been shown to influence the behaviors of endothelia cells.^[^ ^221^ ^]^ Different modes of shear forces, such as laminar, disturbed, or oscillatory flows, regulate endothelial cell functions through multiple sensing mechanisms.^[^ ^222^ ^]^ These mechanical stimuli activate cellular signaling networks, thus affecting gene expressions and functional cellular responses.

Following cell seeding, functional assays are conducted to evaluate the physiological properties of the constructed vascular model. For example, perfusability tests are used to assess lumen integrity and flow continuity;^[^ ^223^ ^]^ barrier integrity assays quantify endothelial cell permeability;^[^ ^224^ ^]^ and shear‐stress responsiveness is evaluated by analyzing cell alignment and junction formation under defined shear conditions.^[^ ^225^ ^]^

### 7.2 Materials: Demand for Intelligent Multifunctionality

In the sacrificial biofabrication, materials are fundamentally categorized into sacrificial materials and surrounding materials, playing an indispensable role in vascular engineering. Despite the remarkable progress achieved with existing materials, they remain inadequate in fully meeting the complex and multifaceted requirements of vascular engineering. This gap underscores an urgent need for the development of next‐generation materials with intelligent multifunctionality to expand the current library, paving the way for more sophisticated and functional vascular constructs.

Sacrificial materials are pivotal because they must not only be processable into well‐defined structures but also be capable of being in situ removed without compromising the integrity of the surrounding matrix. However, despite significant advancements, the current repertoire of sacrificial materials, dominated by polymers and hydrogels, remains limited in meeting the intricate demands of vascular fabrication. A recent breakthrough by using the metallic material, Ga as a sacrificial material, has showcased extraordinary potential.^[^ ^91^ ^]^ Its unique property of being removed via capillary forces enables the creation of complex, multiscale vascular architectures with remarkable precision. This novel material demonstrates the untapped possibilities lying beyond traditional polymeric and hydrogel‐based systems, highlighting the need for pioneering new classes of sacrificial materials. Another challenge is to develop sacrificial materials that possess both high‐resolution printability and the capability to be rapidly and controllably removed under physiological conditions. Our recently proposed DNA hydrogel has demonstrated potential in achieving this delicate balance owing to its unique lubricity and degradability.^[^ ^41^ ^]^ To fully meet the demands of vascular fabrication, a transformative shift towards intelligent sacrificial materials with multifunctionality is required. In addition, to demonstrate that the materials are removed after applying specific stimuli, most studies present microscopic images and perfusion experiments showing the formation of hollow channels, thereby indicating the removal of sacrificial materials. However, the complete removal of sacrificial materials without residues has yet to be explicitly examined. Although this is not essential for some practical applications, residual materials may pose potential long‐term toxicity. Therefore, future studies should investigate whether these sacrificial materials are fully removed.

Surrounding materials constitute the primary structural component, playing a critical role in maintaining the stability and integrity of the overall architecture. In principle, any biomaterial with sufficient mechanical strength, cytocompatibility, and compatibility with both the sacrificial material and its removal method can be utilized as a surrounding material. Current studies are predominantly based on encapsulating tissue cells within the surrounding material, followed by the removal of the sacrificial template to create channels and subsequent endothelial cell seeding to prepare vascularized tissue models. However, native vasculature is highly intricate, multi‐layered structures^[^ ^226^ ^]^ with specialized functional characteristics, including endothelial barrier, contractility, and hemodynamic regulation.^[^ ^5^, ^24^ ^]^ Therefore, incorporating additional supporting cells layer by layer within the surrounding material, such as pericytes, smooth muscle cells, and other perivascular cells, is crucial to accurately replicate the complexity of natural blood vessels and realize their full functional capabilities.

### 7.3 Technologies: Fabrication of Hierarchical Vasculature

Technologies determine the shape of vascular networks by precisely processing sacrificial materials, which are the foundational pillars of the sacrificial biofabrication. Native vascular networks exhibit a complex, hierarchical architecture, spanning from millimeter‐scale conduits to microscale capillaries.^[^ ^24^ ^]^ However, existing fabrication technologies typically limit vascular channel formation to a narrow size range, hindering the ability to accurately replicate the full spectrum of natural vascular complexity.

One of the challenges to replicate native vascular size is enhancing resolution. In human physiology, capillaries are usually below 10 µm in diameter. Yet, except for the spinning technology, most existing technologies fail to achieve the fabrication of 10 µm endothelialized vasculature. Even so, the spinning technology lacks precise control over vascular geometry. This limitation underscores the need for innovative technologies capable of producing controlled high‐resolution endothelialized vasculature. Multiphoton ablation has emerged as a promising technology to address this challenge.^[^ ^89^ ^]^ By leveraging multiphoton excitation to induce localized degradation, this approach enables the direct creation of microchannels within hydrogels in the presence of encapsulated stromal cells.^[^ ^227^ ^]^ This approach holds significant potential for fabricating capillary‐scale vasculature with high precision. In addition to advancing fabrication technologies, optimizing post‐processing methods presents a complementary pathway to enhance vascular resolution. To this end, we have developed a shrinking strategy that utilizes complexation‐induced shrinking of the construct after printing to reduce vascular diameters by 3–8 times, providing a powerful post‐processing solution to achieve superior resolution without altering existing fabrication technologies.^[^ ^86^ ^]^

Another obstacle is the simultaneous construction of both large and small blood vessels within an integrated network. Although some technologies are capable of fabricating vascular channels across a relatively wide diameter range, they remain insufficient to recapitulate the full spectrum observed in native vascular systems. Therefore, multiple fabrication technologies should be integrated to construct fully hierarchical vascular networks. For instance, it is possible to employ 3D printing to produce large vessels, electrospinning to create intermediate‐sized vessels, and in situ self‐assembly to form microvasculatures. By strategically combining these complementary technologies, it becomes possible to engineer vascular networks with full complexity, scalability, and functionality.

## 8 Conclusion

In summary, sacrificial biofabrication shows imperative advantages. Ongoing advancements in materials science and bioengineering are expected to drive further innovations in this field. Future research should focus on optimizing sacrificial materials and surrounding materials, refining printing technologies, and exploring clinical applications to fully realize the potential of this technology. It is believed that this strategy holds significant promise in the field of drug discovery, tissue engineering, and organ regeneration.

## Conflict of Interest

Y.S.Z. consulted for Allevi by 3D Systems; cofounded, consults for, and holds options of Linton Lifesciences; and sits on the scientific advisory board and holds options of Xellar Biosystems. The relevant interests are managed by the Brigham and Women's Hospital. The other authors declare no conflict of interest.
